# Gene expression in term placentas is regulated more by spinal or epidural anesthesia than by late-onset preeclampsia or gestational diabetes mellitus

**DOI:** 10.1038/srep29715

**Published:** 2016-07-11

**Authors:** Tove Lekva, Robert Lyle, Marie Cecilie Paasche Roland, Camilla Friis, Diana W. Bianchi, Iris Z. Jaffe, Errol R. Norwitz, Jens Bollerslev, Tore Henriksen, Thor Ueland

**Affiliations:** 1 Research Institute of Internal Medicine, Oslo University Hospital, Rikshospitalet, Oslo, Norway; 2 Mother Infant Research Institute, Tufts Medical Center, Boston, MA, USA; 3 Department of Medical Genetics, Oslo University Hospital and University of Oslo, Oslo, Norway; 4 Department of Obstetrics, Oslo University Hospital, Rikshospitalet, Oslo, Norway; 5 Molecular Cardiology Research Institute and Division of Cardiology, Department of Medicine, Tufts Medical Center, Boston, MA, USA; 6 Department of Obstetrics & Gynecology, Tufts Medical Center and Tufts University School of Medicine, Boston, MA, USA; 7 Faculty of Medicine, University of Oslo, Oslo, Norway; 8 Section of Specialized Endocrinology, Department of Endocrinology, Oslo University Hospital, Rikshospitalet, Oslo, Norway

## Abstract

Pre-eclampsia (PE) and gestational diabetes mellitus (GDM) are common complications of pregnancy, but the mechanisms underlying these disorders remain unclear. The aim was to identify the extent of altered gene expression in term placentas from pregnant women with late-onset PE and GDM compared to controls. RNAseq identified few significantly differentially regulated genes in placental biopsies between PE, GDM, or uncomplicated pregnancy (n = 10 each group). Five genes were altered in placentas from PE including 4 non-coding genes and Angiopoietin 2 (*ANGPT2*). No genes were significantly regulated by GDM. In contrast, many genes were significantly regulated by fetal, maternal and delivery-specific variables, particularly spinal and epidural anesthesia. We selected *ANGPT2* and Chemokine (C-X-C motif) ligand 14 (*CXCL14*) to test with qPCR in a larger set of placentas (n = 475) and found no differences between the groups. However, regression analysis revealed a stronger association between placental *ANGPT2* and *CXCL14* mRNA expression and fetal, maternal and delivery-specific variables than diagnostic group. To conclude, the gene expression in term placentas are highly affected by fetal, maternal and delivery specific variables. Few regulated genes were found in late-onset PE and GDM placentas, which may suggest that these conditions could be more affected by maternal factors.

The placenta is a composite structure of embryonic and maternal tissues that supplies nutrients and removes waste products from the developing embryo. It also has critical endocrine and immuno-modulatory properties. Up to 10% of pregnancies suffer from abnormal placental development and, as such, are at high risk for complications such as miscarriage, pre-eclampsia (PE), gestational diabetes mellitus (GDM), fetal growth restriction and preterm birth^1^.

Pre-eclampsia is characterized by new-onset hypertension and end-organ damage after 20 weeks of gestation and affects 3–5% of all pregnancies^2^. PE has a multifactorial etiology and includes several subtypes^3^. Early-onset PE generally involves poor placental development, whereas late-onset PE is believed to arise from the interaction between a normal placenta and a damaged maternal microvasculature due, for example, to chronic hypertension or diabetes^4^. The late-onset form of PE may thus be more similar to GDM, in which pregnancy serves as a stress test in predisposed women^5^,^6^. GDM is defined as glucose intolerance of variable degrees first recognized during pregnancy. It may affect up to 20% of pregnant women depending on ethnicity and the diagnostic criteria used^7^.

The exact mechanisms behind GDM and PE are unclear. Previously, PE and other placental disease-related complications were considered pregnancy-specific and were thought to resolve hours or days after delivery of the placenta. More recently it has become clear that such diseases may contribute to the development of future cardiovascular disease^8^,^9^, providing additional need to understand the role of the placenta in the etiology of these disorders. Thus, identifying genes critical for placenta function may serve as a basis for revealing mechanisms underlying both normal and pathologic pregnancies. Gene expression in PE placentas has been extensively studied using microarrays^10^,^11^,^12^ and RNA sequencing (RNAseq)^13^,^14^ and to a lesser extent in placentas from GDM women^15^,^16^,^17^. However, the differentially expressed genes (DEGs) identified in these studies vary greatly, likely due to the phenotypic and etiologic heterogeneity of PE and the different diagnostic criteria used for GDM. Furthermore, some factors that may affect placental gene expression and add further heterogeneity between the studies (such as offspring sex and delivery specific variables)^14^ are rarely acknowledged or accounted for.

This study investigates the potential of RNAseq to discover novel genes that may encode the prediction and management of PE and GDM. Our aims were: (1) to examine DEGs in placentas from pregnant women with late-onset PE and GDM compared to normal placentas by RNAseq; (2) to identify DEGs in relation to maternal, fetal and delivery-specific variables by RNAseq; and (3) to validate our DEGs identified by RNAseq using RT-qPCR in a larger sample set and to control for the influence of maternal, fetal and delivery-specific variables.

## Results

### Clinical characteristics

Table 1 shows the characteristics of the study population chosen for RNAseq (n = 30) and validation with RT-qPCR (n = 475). In the RNAseq cohorts, as expected, women with GDM had a significantly higher BMI and placental weight and women with PE had a higher DBP at visit 4. In the validation cohort, GDM and PE women had higher BMI, BP, more frequent induction of labor, and shorter duration of labor. In addition, GDM women were older, had higher birth weight and placental weight, had more cesarean deliveries, and were more often nulliparous. Gestational age was shorter in PE women. Women with PE had no indication of poor placental function based on birth weight and the relatively long gestational age underlining the mildness of the phenotype. Blood pressure in the PE patients were higher than 140/90, but we did not have access to exact values after visit 4, and we could therefore not include severity of PE women based on blood pressure. Using World Health Organization (WHO) as opposed to International Association of Diabetes and Pregnancy Study Groups (IADPSG) criteria for the diagnosis of GDM, the differences with controls were somewhat attenuated (Supplemental Table 1). Supplemental Table 2 compares some maternal, fetal and delivery specific variables between women with and without epidural or spinal anesthesia demonstrating that these groups were comparable for most variables. However, a higher umbilical cord venous base excess and a higher incidence of cesarean deliveries were observed in the spinal group compared to women without anesthesia.

### Highest expressed genes in term placentas

**Figure 1:**
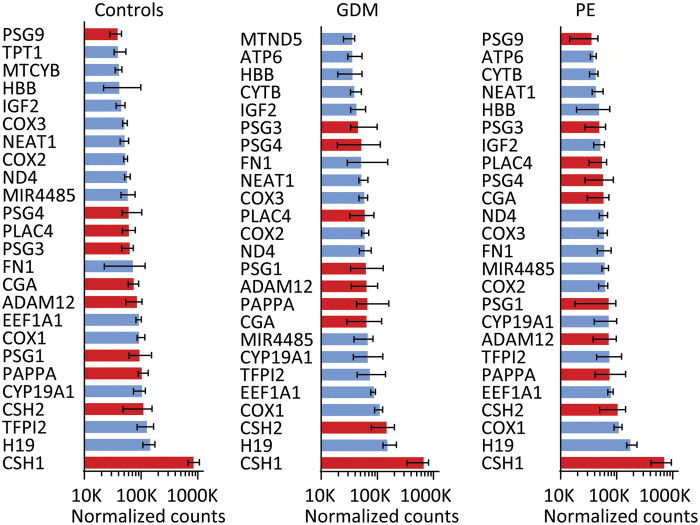
Top 25 expressed transcripts in the term placenta for controls, GDM, and PE. Data are given as median normalized counts for and 25^th^/75^th^ percentile, and are shown on a logarithmic scale. Transcripts with exclusive expression in placental tissue are represented by red columns.

The genes were ordered by their median expression level. The highest expressed genes were similar in normal, GDM and PE placentas (Fig. 1). Genes that may regulate placental/fetal growth were highly expressed, including the noncoding RNA, H19, and insulin growth factor 2 (*IGF2*)^18^. Many placental-specific genes were also highly expressed, i.e. the placental lactogens (*CSH1* and *CSH2*), placenta specific glycoproteins (*PSG1*, *PSG3* and *PSG4*, *PSG5* and *PSG9*), pregnancy-associated plasma protein A (*PAPPA*), hCG alpha subunit (CGA), estrogen synthase (*CYP19A1*), ADAM metallopeptidase domain 12 (*ADAM12*), tissue factor pathway inhibitor 2 (*TFPI2*), and placenta specific 4 (*PLAC4*). Other highly expressed genes were fibronectin (*FN1*), NADH dehydrogenase subunit 4 (*ND4*), nuclear paraspeckle assembly transcript 1 (*NEAT1*), eukaryotic translation elongation factor 1 alpha 1 (*EEF1A1*), cytochrome c oxidase genes (*COX1*, *COX2* and *COX3*), cytochrome b (*CYTB*), hemoglobin beta (*HBB*) and microRNA 4485 (*MIR4485*).

The amount of RNA coming from maternal decidual and nucleated blood cells was examined by looking at the *XIST* transcript in the XX (median count 10099) and XY (median count 218.5) placenta samples. Median estimate for the fraction of RNA originated from maternal cells was 2.2% range (25^th^, 75^th^) 1.7–4.6% (mean, 4.0%). This suggests that the RNAseq data was of good quality both technically and biologically with little contamination from the maternal decidua or blood^14^.

### Effects of maternal, delivery-specific, and fetal variables on placental gene expression by RNAseq

**Figure 2:**
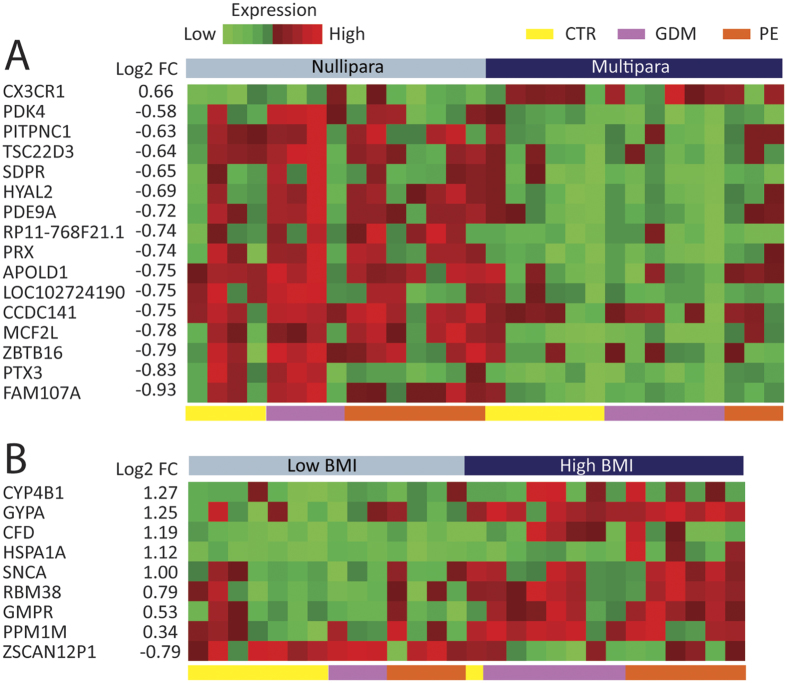
Effect of maternal variables on placental gene expression. A heat map is shown demonstrating the effect of two maternal variables on placental DEGs: (**A**) parity (nulliparous vs. multiparous), and (**B**) BMI (low-median vs. high) with FDR < 0.1. Heatmaps were constructed using log2 expression levels. The numbers to the left shows the log2 fold change value (log2FC) between groups. Samples are shown at the bottom of the figure, controls (yellow [CTR]), GDM (purple), and PE (red).

We evaluated potential maternal, delivery-specific, and fetal confounding variables on placental DEGs with FDR < 0.1. Investigating maternal confounding variables using all 30 samples, 16 DEGs were detected between primiparous compared to multiparous women. Of these, 15 were downregulated and 1 was upregulated in the multiparous women (Fig. 2). Some of these genes are involved in placentation and inflammation, i.e. pentraxin 3 (*PTX3*), chemokine (C-X3-C motif) receptor 1 (*CX3CR1*), and TSC22 domain family, member 3 (*TSC22D3*). Nine DEGs were detected between low and high BMI patients at visit 4. Of these, 8 were upregulated and 1 downregulated in the placenta from women with higher BMI, including increased adipsin (*CFD*). For BMI visit 1 and age of the mother, we did not find any significantly DEGs, FDR < 0.1 (data not shown).

**Figure 3:**
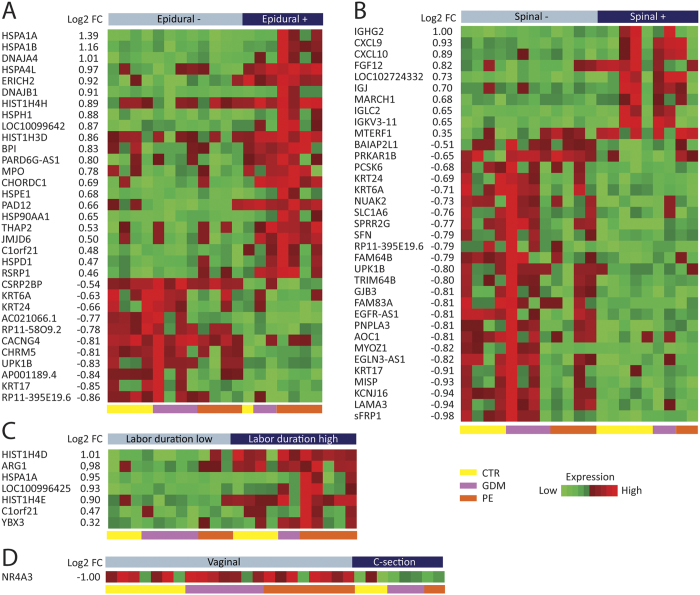
Effect of delivery-specific variables on placental gene expression. A heat map is shown demonstrating the effect of four delivery-specific variables on placental DEGs: (**A**) epidural anesthesia (yes vs. no), (**B**) spinal anesthesia (yes vs. no), (**C**) labor duration (low-median vs high), and (**D**) mode of delivery (vaginal vs. cesarean) with FDR < 0.1. Heatmaps were constructed using log2 expression levels. The numbers to the left shows the log2 fold change value (log2FC) between groups. Samples are shown at the bottom of the figure, controls (yellow [CTR]), GDM (purple), and PE (red).

For the delivery-specific variables (Fig. 3), we found 33 DEGs with epidural anesthesia (22 upregulated and 11 downregulated) and 35 DEGs with spinal analgesia (10 upregulated and 25 downregulated). Women who received general anesthesia (n = 1) and pudendal block (n = 1) were excluded from these analyses. Some DEGs were similar between epidural and spinal anesthesia (e.g., keratins), but there were also specific differences in the gene signature depending on the anesthesia type. In particular, 9 transcripts for heat shock proteins (Hsp) belonging to the Hsp70 and Hsp40 family were abundantly expressed and increased in placentas from women who received epidural anesthesia. In addition, myeloperoxidase (*MPO*), a marker of neutrophil activation, was enhanced in women who received epidural anesthesia. In women who received spinal anesthesia, genes involved in the regulation of immune responses including several immunoglobins (e.g. *IGHG2, IGJ, IGCL2, MARCH1*) and chemokines (*CXCL9/10*) were differentially increased. With regard to differences relating to type of delivery, 1 DEG was downregulated in the women who delivered by cesarean, and 7 DEGs were upregulated in the placentas of women with a longer duration of labor, while no DEGs were associated with induction of labor.

**Figure 4:**
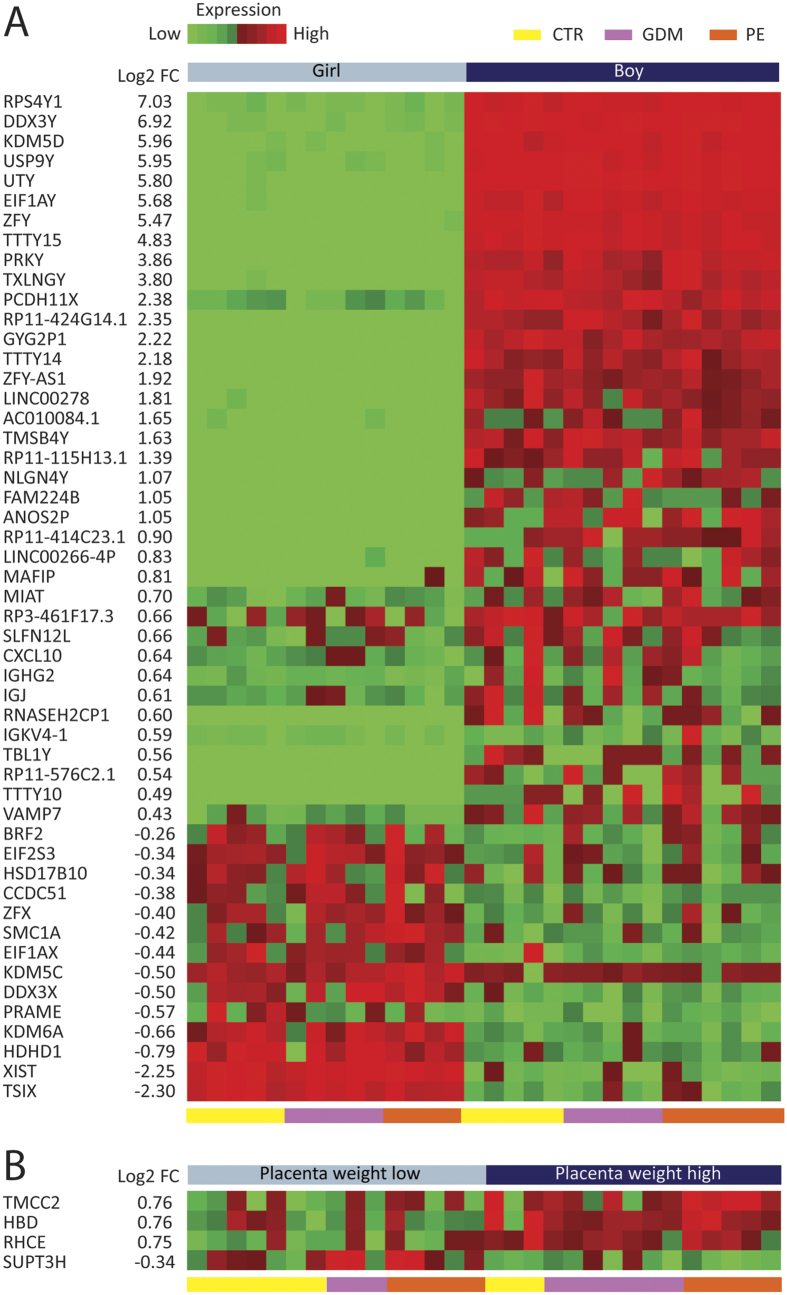
Effect of fetal variables on placental gene expression. A heat map is shown demonstrating the effect of three fetal variables on placental DEGs: (**A**) Offspring sex (female vs. male), and (**B**) placenta weight (low-median vs. high) with FDR < 0.1. Heatmaps were constructed using log2 expression levels. The numbers to the left shows the log2 fold change value (log2FC) between groups. Samples are shown at the bottom of the figure, controls (yellow [CTR]), GDM (purple), and PE (red).

For fetal confounding variables (Fig. 4), 51 DEGs were associated with offspring’s sex. Thirty-seven were upregulated and 14 were downregulated when using males as the reference. Most of these were sex-specific chromosomal genes. We found 4 DEGs between the median high and low placental weights. Three were upregulated and 1 was downregulated in those with the highest placental weight. For birth weight, no significant DEGs were observed (Fig. 4). A list of the 70 most DEGs for the confounding variables can be found in Supplemental Table 3.

### Effect of PE and GDM on placental gene expression by RNAseq

Only 5 DEGs were identified between the control and PE placentas with a FDR < 0.1. Of these, there were 3 downregulated non-coding genes, one downregulated protein-coding gene (Angiopoietin 2 [*ANGPT2*]), and one upregulated non-protein coding gene. Median counts (25^th^–75^th^ percentile) for *ANGPT2* in control *vs*. PE placentas were 663 (224–933) and 239 (155–386), respectively. No DEGs were detected between GDM and control placentas with FDR < 0.1. The 70 most DEGs for the diagnostic groups based on FDR and unadjusted p-value are listed in Supplemental Table 4.

### Validation of RNAseq data by RT-qPCR

To validate our RNAseq data, we performed RT-qPCR. Samples for RT-qPCR validation included not only the 30 samples included in the RNAseq analysis, but all 475 placental samples from the STORK study with high RNA quality. However, since few genes were differentially expressed, in addition to *ANGPT2*, we chose to measure *CXCL14* which was on the top of the lists for both PE and GDM (unadjusted p-value < 0.0006 and <0.001 respectively), and may be known for potentially regulate trophoblast outgrowth at the maternal-fetal interface^19^. As seen in Supplemental Fig. 3, differential (decreased) expression of *ANGPT2* in PE placentas was confirmed by qPCR in the same 30 samples used in the RNAseq experiment. However, in the larger sample set, although *ANGPT2* expression was decreased in PE placentas compared with controls, the difference did not reach statistical significance (P = 0.74). *CXCL14* expression was not increased by qPCR in GDM (p = 0.055) and PE (p = 0.085) placentas in the same 30 samples used in the RNAseq experiment or in the larger sample set (p = 0.14 (GDM WHO), p = 0.11 (GDM IADPSG), p = 0.93 (PE)) (Supplemental Fig. 3).

### Prediction of *ANGPT2* and *CXCL14* expression in placenta in the large cohort

To evaluate the influence of maternal, delivery- specific, and fetal characteristics on *ANGPT2* and *CXCL14* expression, we finally performed multivariable regression analysis, including diagnostic group (i.e. GDM or PE) to identify the most important predictors (Table 2). Stepwise linear regression identified gestational age, parity and offspring sex as predictors of *ANGPT2* expression. Gestational age followed by birth weight and parity were the variables that predicted the gene expression of *CXCL14*.

## Discussion

We conducted RNAseq analysis to compare gene expression between late-onset PE, GDM and control placentas at term. Using this approach, few genes were differentially expressed between these groups. In contrast, maternal, delivery-specific, and fetal variables were associated with altered expression of multiple genes in the term placenta. In particular, spinal and epidural anesthesia were unexpectedly associated with a significant upregulation of heat shock protection and immunomodulatory genes. When using qPCR in a larger cohort to validate the most highly differentially regulated genes in late-onset PE and GDM placentas identified in the RNAseq experiment (namely, *ANGPT2* and *CXCL14*), we noted that maternal, delivery-specific, and fetal variables were stronger predictors of gene expression than the diagnostic group. These results suggest that maternal, delivery, and fetal variables substantially contribute to placental gene expression and should be considered when designing and evaluating such studies.

Samples chosen for RNAseq were well matched by clinical criteria and the RNA was of acceptable quality with a small percentage of contamination by maternal decidual and nucleated blood cells (~2%). This is similar to data published by Sõber *et al*. following a single wash with phosphate buffer saline and stabilization in RNA-later solution^14^. Furthermore, the most abundantly expressed genes identified in the term placenta were ones known to be involved in placental function, with good overlap with transcripts identified in previous studies^14^. For the sex specific genes, 15 and 19 of the DEGs identified in the current study corresponded with prior publications by Sõber *et al*.^14^ and Buckberry *et al*.^20^, respectively. Other DEGs associated with offspring sex in the current study were either non-functional genes expressed at low levels or sex specific genes (i.e. *TSIX, TXNLGY, DDX3X, TTTY14, TBL1X*, *TBL1Y* and *TTTY10*) not reported in the prior manuscripts. Totally from the 51 DEGs, 28 were located on the Y-Chromosome while 12 were located on the X-Chromosome. Taken together, the similarities in gene expression, related to abundance and offspring sex, with previous studies as well as the low maternal contamination supports the validity of our placental RNA samples.

A major finding in this study was the significant impact of epidural and spinal anesthesia on placental gene expression. To our knowledge, this has not been previously reported. Since women receiving epidural may be more likely to experience hyperthermia^21^, the increased expression of mRNAs coding for members of the HSP family could reflect the heat shock response, a switch in transcription and translation to preferentially express HSP’s that may preserve essential proteins^22^. Other relevant causes of increased HSP expression due to different kinds of stress could be infection, inflammation, hypoxia, injury and also adrenaline, which is a component in epidural anesthesia^23^,^24^. In particular, we identified a higher expression of the genes encoding HSP70, a HSP frequently studied in placental disease that may also confer harmful effects on binding to toll-like receptors^25^. Of note, increased expression of HSP70 protein has been demonstrated in the placenta of women with PE^26^,^27^ and in relation to delivery-specific variables, such as preterm labor^28^. In contrast, we found no significant association between HSP expression and mode of delivery, induction and duration of labor, or presence of PE. Several studies suggest that underlying maternal inflammation may confer increased risk of fever following epidural anesthesia^29^,^30^. However, apart from increased *MPO* mRNA levels in women receiving epidural, possibly reflecting neutrophil activation during the hyperthermic response^31^, few inflammatory genes were differentially regulated. In contrast, the use of spinal anesthesia was associated with enhanced mRNA expression of several inflammatory genes, including *IGJ, CXCL9* and *CXCL10*, all chemokines specific for T cell signaling through the CXCR3 receptor. Activation of CXCR3 signaling has been implicated in the onset of labor^32^. Raman *et al*. identified enhanced IGJ expression as the most distinguishable feature of chronic placental inflammation in addition to CXCL9 and other inflammatory components from T and B cells^33^. Activation of opioid receptors increases the expression of CXCL10 in circulating immune cells *in vitro*^34^ and in the spinal cord in experimental animal models *in vivo*^35^. The use of anesthesia may reflect underlying obstetric conditions and we cannot excluded that the differences in mRNA expression observed for use of epidural and spinal anesthesia may results from longer acting mechanisms and systemic stress responses of the mother.

For maternal confounding variables, the expression of genes involved in inflammation and placentation (i.e., *CX3CR1*^36^, *TSC22D3*^37^ and *PTX3*)^38^ were associated with parity, indicating that women who have had a prior pregnancy may respond differently to the presence of invading trophoblast compared to nulliparous women. Adipsin expression, which is shown to be abundant in adipose tissue, was increased in the placentas of women with high BMI at term, and placentas from obese women have been shown to secrete higher levels of adipsin^39^.

Surprisingly few genes were differentially expressed in the placentas of women with late-onset PE at term and none in the placentas of women with GDM. We found only five significantly differentially regulated genes in PE placentas based on FDR < 0.1. The result of two recent meta-analyses found few common genes regulated in PE placentas compared to controls^10^,^12^. Using unsupervised clustering of 7 microarray data sets, Leavey *et al*.^3^ found a large degree of co-clustering of PE and control samples suggesting no distinct gene signature in PE. Direct comparison with previous studies is difficult since these mainly have focused on early-onset PE, a separate entity from late-onset PE as demonstrated in a recent micro-array study^40^. Furthermore, Sõber *et al*. identified an extensive shift in the placental transcriptome profile in late-onset PE using RNAseq^14^. For one specific DEG, ZDHHC8P1, we found increased expression in late-onset PE patients which were in contrast to their results were it was downregulated in their late-onset PE. However, their patients had a markedly more severe phenotype according to the ACOG 2013 criteria (hypertension with additional symptoms^41^ compared to the milder PE phenotype in our patients. This could imply that milder forms of preeclampsia that meet diagnostic criteria may in fact involve no underlying placental pathology and therefore may technically represent gestational hypertension with concomitant kidney dysfunction. It’s possible these cases may respond well to standard antihypertensive therapy and not require the termination of pregnancy.

As for GDM, previous studies evaluating gene expression have been performed in mixed cohorts (i.e. women with both PE in addition to GDM)^15^, in women on insulin therapy^15^,^17^, using different diagnostic criteria, or with very small sample numbers^16^, making comparisons difficult. Since our patients were not on insulin, it may be reasonable to assume that they have a milder GDM phenotype compared to other studies^15^,^17^. Importantly, excluding some differences in BMI and placental weights (Table 1), the major confounding maternal, delivery-specific and fetal variables were similar between our three comparison groups (late-onset PE, GDM and controls). Furthermore, when validating *ANGPT2* and *CXCL14* gene expression in the larger cohort, maternal, fetal and delivery-specific covariates were by far the strongest determinants of their expression. Since the objective of expression analysis is to identify unique expression patterns for distinct disease entities and not necessarily the effect of confounding conditions, our results highlight the importance of well characterized phenotypes to allow for accurate matching and relevant comparison between groups.

Several limitations of this study should be emphasized. The quality of the RNA could have been better. Five out of 30 RNAseq samples had RIN values below 7, and additional 8 samples had values below 8. However, we judged them to be of acceptable quality as judged by electropherograms. The placenta is a heterogeneous tissue and the biopsy was taken from the placental parenchyma. Other parts and specific cells of the placenta may be more important when investigating genes regulated in PE and GDM. Only one biopsy was taken from each placenta and the collection was performed by different individuals. While the technique for placental biopsy was standardized, we cannot exclude the possibility that biopsies were taken at different sites and that regional differences may have accounted for differences in DEGs^42^. Also, only term placentas were used in our analysis. While this ensures that appropriate control placentas will be available for comparison, it does represent the endpoint of a chronic disease state. It is likely that mRNA expression profiles at an earlier stage will be more reflective of the origin and pathogenesis of the disease and may be more likely to yield a distinctive fingerprint. This is especially true of PE^3^. Also, as we studied late-onset PE, our results cannot be applied to the spectrum of PE. Finally, we have incomplete information about the precise timing from when the placenta was delivered to when the biopsy was taken and the tissue stored at −80 °C.

In conclusion, the current study shows that confounding variables have a greater influence on gene expression in term placenta than do underlying disease states (such as late-onset PE and GDM). We found few genes significantly differentially regulated between late-onset PE, GDM and control placenta, suggesting that maternal factors may be more important than placental (fetal) factors, in the genesis of these pregnancy-specific conditions.

## Methods

### Study population

The STORK study was a prospective cohort study with a longitudinal design in which 1031 low-risk women of Scandinavian heritage who planned to deliver at Oslo University Hospital, Rikshospitalet, between 2002 and 2008 were followed throughout their pregnancy. Details about the study have been previously published^43^. Exclusion criteria included multiple pregnancy, known pre-gestational diabetes, severe chronic medical conditions (such as lung, cardiac, gastrointestinal or renal diseases), and pregnancies complicated by major fetal malformations or aneuploidy. Patients were routinely monitored for the development of pregnancy complications, such as PE, GDM, or preterm labor. Demographic and clinical data (including use of anesthesia in labor, route and mode of delivery, and neonatal outcome) were abstracted from the medical records. The difference in the formula used at our hospital between the epidural and spinal anesthesia is adrenaline in the epidural anesthesia, in addition to opioids, both fentanyl (or sufentanil in spinal) and bupivacaine in both.

Written informed consent was obtained from all participants. All clinical investigations were conducted according to the principles in the Declaration of Helsinki. The study was approved by the Regional Committee for Medical Research Ethics of Southern Norway in Oslo, Norway.

### Clinical Diagnosis of GDM

A 75 g oral glucose tolerance test (OGTT) was performed in the morning after an overnight fast on all women at 30–32 weeks of gestation. Venous blood samples collected into tubes containing EDTA were analyzed at point of care using an Accu-Check Sensor glucometer (Roche Diagnostics GmbH, Mannheim, Germany). Additional venous blood samples were allowed to clot for 30 min. The serum was separated by centrifugation for 10 min at 3000 *g* and stored at −80 °C. Glucose levels were also measured from frozen serum samples collected at 30–32 weeks using the hexokinase method (Hitachi Modular P800, Roche Diagnostics, Mannheim, Germany) at an accredited clinical chemistry laboratory at Oslo University Hospital Rikshospitalet, as previously reported^44^. GDM was diagnosed on a 75 g OGTT using both the new IADPSG criteria and the old WHO criteria as follows: (1) IADPSG criteria: fasting plasma glucose (FPG) of 5.1–6.9 mmol/L (92–124 mg/dL) and 1 h plasma glucose ≥10.0 mmol/L (≥180 mg/dL) or 2 h plasma glucose 8.5–11.0 mmol/L (153–198 mg/dL); and (2) WHO criteria: 2 h plasma glucose ≥7.8 mmol/L (140 mg/dL)^45^, as previously reported^44^.

### Clinical Diagnosis of PE

PE was diagnosed by new-onset blood pressure ≥140/90 mmHg and significant proteinuria (urinary total protein/creatinine ratio >30 or +1 on urine dipstick). All cases were diagnosed after 34 weeks gestation (late-onset PE).

### Collection, storage and RNA extraction of placental biopsies

Placental biopsies were collected after vaginal or cesarean delivery. One biopsy was taken per placenta. The STORK study set included 475 placental samples, with good RNA quality, in which 19 were from women with PE, 103 with GDM, and 353 controls (overview, Supplemental, Fig. 1). Blocks of 2–4 cm were taken from the placental parenchyma, briefly washed in phosphate buffer saline, snap frozen in liquid nitrogen, and stored at −80 °C until RNA isolation. Half of the biopsy was homogenized in TRIzol reagent (Invitrogen, Life Technologies) on ice with a tissue grinder (Sigma Aldrich). Total RNA was extracted using TRIzol reagent (Invitrogen, Life Technologies) and purified with RNeasy microkit columns (Qiagen, Netherlands). Purity and concentration of isolated total RNA was measured using Nanodrop ND-1000 Spectrophotometer (Thermo Fisher Scientific Inc., USA) and RNA integrity number (RIN) was estimated using Agilent 2100 Bioanalyzer (Agilent Technologies, USA). RNA samples with a RIN <4 were excluded from subsequent RNAseq and qPCR analysis^46^.

### RNAseq

Samples with a RIN greater than 4 were preferred for RNAseq analysis (median RIN 8.6; range (25^th^, 75^th^), 7.9–9.2). The RIN values and gel/electrophoresis results from the samples used for RNAseq are shown in Supplemental Fig. 2. The samples chosen for RNAseq were matched (based on mothers age and gestational age) between the groups of normal (control), GDM pregnancy and pre-eclamptic pregnancy (10 samples in each group). Sequencing libraries were prepared from 500 ng of total RNA using the TruSeq RNA sample preparation reagents (Illumina, San Diego, California) according to the manufacturer’s instructions, with fragmentation for 4 minutes at 94 °C. The libraries were sequenced using 125 bp paired-end sequencing on an Ilumina HiSeq 2000. We recorded an average 22.3 million (range, 20.2–24.6 million) paired reads per sample. Fastq files were generated using bcl2fastq (v1.8.4). Sequence reads were mapped to the reference genome (hg19/GRCh38) using TopHat2 (v2.0.13) and Bowtie2 (v.2.2.3.0). Library sizes and standard deviations for input into TopHat were calculated empirically by aligning 1000000 reads to an index built from human cDNA sequence. Sequence alignment was guided using only previously annotated gene models downloaded from Ensembl (www.ensembl.org; Homo_sapiens.GRCh38.79.gtf). On average, there was 73.6% concordant read pair mapping (range 69.6–76.6), with a mean unique mapping of 94.1%. Raw expression counts were calculated per gene using featureCounts (http://bioinf.wehi.edu.au/featureCounts/) and the same gtf file which was used for the read alignment.

Differential expressed genes in RNAseq data was tested using DESeq2^47^ package for R. Outlier detection (Cook distance cutoff) and filtering out low expressed genes was performed using the default method in DESeq2. Example code is provided below:

# DE - control v preeclampsia

sampleTableCvP <- read.csv(“/SampleSheet_control_Preeclampsia.csv”)

countTableCvP <- read.table(“counts_pBs2_control_Preeclampsia.txt”)

countMatrixCvP <- as.matrix(countTableCvP)

ddsCvP <- DESeqDataSetFromMatrix(countData = countMatrixCvP, colData = sampleTableCvP, design = ~ Status)

ddsCvP$Status <- relevel(ddsCvP$Status, “control”)

ddsCvP <- DESeq(ddsCvP)

(resCvP <- results(ddsCvP))

For the highest expressed genes in the control, GDM and PE group we normalized the counts by this example code:

sampleTableAll <- read.csv(“SampleSheet.csv”)

countTableAll <- read.table(“counts_pBs2.txt”)

countMatrixAll <- as.matrix(countTableAll)

ddsAll <- DESeqDataSetFromMatrix(countData = countMatrixAll, colData = sampleTableAll, design = ~ Status)

vsd <- varianceStabilizingTransformation(ddsAll, blind = FALSE)

write.table(assay(vsd), “vsd.txt”, quote = F, sep = “\t”)

### Maternal, Delivery-specific and Fetal Confounding factors

The confounding variables investigated in the RNAseq of the 30 placenta samples were dichotomized by median low *vs*. high or divided by categories (Table 1). Maternal variables: parity (nulliparous *vs.* multiparous), age (median 31 years, 25-median (low) *vs.* median-42 (high)), BMI visit 1 (median 24.9 kg/m^2^, 17.2-median (low) *vs.* median-34.9 (high)) and 4 (median 28.3 kg/m^2^, 20.5-median (low) *vs*. median-38.1 (high). For delivery specific variables, we investigated anesthesia (spinal *vs*. no anesthesia) and (epidural *vs*. no anesthesia), delivery mode (vaginal *vs*. cesarean section), duration of labor (median 5 hours, 2-median (low) vs. median-15 (high) and induction of labor (oxytocin or prostaglandin *vs*. no induction). For fetal variables we investigated offspring sex (girls *vs*. boys), placental weight (median 700 g, 530-median (low) *vs*. median-1100 (high)), and birth weight (median 3610 g, 2910-median (low) *vs*. median-4760 (high)). Gestational age had a very narrow spectrum (median 39.9 weeks, (37.7- median (low) *vs*. median-41.1 (high)) to make a meaningful analysis and was excluded from the RNAseq confounding factors analysis.

### RT-qPCR

Reverse transcription was performed using a High Capacity cDNA Archive Kit (Applied Biosystems, Foster City, CA) with 1 μg of total RNA. mRNA quantification was performed using Power SYBR Green PCR Master Mix (Applied Biosystems, Foster City, CA) and the standard curve method on an ABI Prism 7900 (Applied Biosystems) with duplicate samples and standards. RT-qPCR of select genes was then performed on RNA extracted from all 475 placental samples with acceptable quality in the entire STORK population (n = 475, which included 19 with PE, 103 with GDM, and 353 controls) to validate the RNAseq results. In brief, sequence specific intron spanning oligonucleotide primers were designed using the Primer Express software version 2.0 (Applied Biosystems). *ANGPT2* ‘GACACACCACGAATGGCATCTA (forward), GGGTTACCAAATCCCACTTTATATT’ (reverse), and *CXCL14* GGACGGGTCCAAATGCAAGT (forward), GGTACCTGGACACGCTCTTG (reverse). Transcript expression levels were normalized to the geometric mean of three reference genes known to have great expression stability in placentas^48^; *YWHAZ* GATGACAAGAAAGGGATTGTCGAT (forward), CAGACCCAGTCTGATAGGATGTGT (reverse), *TBP* GCAGCTGCAAAATATTGTATCCACA (forward), CGTGGTTCGTGGCTCTCTTA (reverse), *SDHA* TCCTGATGGAGAATGGGGAGT (forward), GACGTGCAGCTGAAGTAGGT (reverse) and expressed as relative mRNA levels (data were log transformed to normal distribution).

### Statistical analyses

Statistical analyses were conducted using SPSS for Windows, version 21.0 (Chicago, IL, USA). Data are expressed as mean ± SD when normally distributed and median (25^th^, 75^th^ percentile) when skewed. Comparison between women with GDM or PE compared to controls was performed using t-test or Mann-Whitney U, depending on distribution, and Chi-square test or Fisher’s exact test (less than five observations) for categorical variables. Univariate and stepwise (variables p < 0.2 in univariate analysis) linear regression analyses were carried out on log transformed variables (if skewed) and results given as standardized regression coefficients. Linear relationship, multivariate normality, multicollinearity tested by variance inflation factors, autocorrelations tested by Durbin-Watson and homoscedasticity were tested and found satisfactory for linear regression. A gene in RNAseq was considered as differently expressed when the false discovery rate (FDR) was <0.1. FDR was calculated according to Benjamini and Hochberg.

## Additional Information

**How to cite this article**: Lekva, T. *et al*. Gene expression in term placentas is regulated more by spinal or epidural anesthesia than by late-onset preeclampsia or gestational diabetes mellitus. *Sci. Rep.* **6**, 29715; doi: 10.1038/srep29715 (2016).

## Supplementary Material

Supplementary Information

## Figures and Tables

**Table 1:** Maternal, fetal and delivery specific variables in women with normal pregnancies (Ctrl), gestational diabetes (GDM, IADPSG) and preeclampsia (PE) in the RNAseq (n = 30) and RT-qPCR (n = 475) cohorts.

Characteristics	RNAseq			RT-qPCR		
	Ctrl (n = 10)	GDM (n = 10)	PE (n = 10)	Ctrl (n = 353)	GDM (n = 103)	PE (n = 19)
Maternal						
Age (years)	31.9 ± 4.4	32.6 ± 5.4	30.3 ± 3.2	31.4 ± 3.9	32.3 ± 4.4**	30.4 ± 3.8
BMI visit 1 (kg/m^2^)	22.6 (20.2, 24.9)	27.1 (24.9, 30.4)*	26.6 (22.8, 31.4)	23.8 (21.6, 25.8)	26.0 (23.5, 28.8)**	28.6 (24.5, 31.4)**
BMI visit 4 (kg/m^2^)	25.7 (23.4, 27.2)	29.3 (28.2, 33.7)*	30.0 (26.5, 35.2)	27.4 (25.0, 29.7)	29.0 (27.1, 32.0)**	32.6 (27.3, 35.4)**
Smokers^a^ n (%)	1 (10)	3 (30)	1 (10)	73 (20)	29 (30)	3 (16)
Primipara^b^ n (%)	3 (33)	4 (40)	7 (70)	191 (55)	42 (42)*	13 (68)
SBP visit 1 (mmHg)	110 (100, 120)	113 (110, 136)	113 (110, 120)	110 (100, 115)	115 (105, 120)**	120 (110, 120)**
SBP visit 4 (mmHg)	113 (110, 119)	115 (108, 135)	130 (118, 141)	110 (105, 120)	115 (110, 120)**	130 (120, 138)**
DBP visit 1 (mmHg)	70 (60, 70)	70 (60, 76)	70 (65, 80)	65 (60, 70)	70 (60, 75)*	70 (65, 80)*
DBP visit 4 (mmHg)	70 (63, 75)	73 (70, 83)	83 (79, 96)**	70 (65, 80)	70 (65, 80)	83 (76, 90)**
Delivery specific						
Anesthesia (spinal, epidural, pudendal, general), n (%)	5,1,0,0 (50,10,0,0)	2,2,1,1 (20,20,10,10)	2,4,0,0 (20,40,0,0)	57,149,14,3 (16,42,0,1)	26,27,3,2 (25,36,2,1)	4,8,1,0 (21,42,5,0)
Cesarean delivery, n (%)	3 (30)	3 (30)	2 (20)	52 (10)	29 (27)**	5 (26)
Duration of labor (hours)^c^	10.0 (4.0, 13.0)	4.0 (3.0, 7.0)	5.5 (2.3, 9.3)	7.0 (4.0, 11.0)	5.0 (3.0, 8.0)**	3.8 (2.0, 7.0)*
Induced labor, n (%)^d^	0 (0)	2 (29)	5 (50)	58 (18)	30 (35)**	11 (65)**
Fetal						
Gestational age (weeks)	39.9 (38.7, 40.6)	39.6 (38.7, 40.2)	40.1 (39.4, 40.3)	40.4 (39.4, 41.0)	40.0 (39.0, 41.0)	39.7 (38.6, 40.3)**
Birth weight (g)	3601 ± 498	3834 ± 454	3664 ± 597	3624 ± 482	3795 ± 502**	3698 ± 589
Female (%)	5 (50)	5 (50)	4 (40)	157 (48)	54 (52)	11 (58)
Placental weight (g)	700 (630, 785)	890 (705, 943)*	675 (587, 736)	700 (610, 800)	750 (630, 900)*	700 (610, 760)

Data given as mean ± SD when normal distributed and median (25^th^, 75^th^) when skewed distributed. Comparison between placentas from women with GDM and controls, and PE and controls, were performed using t-test for normal distributed variables, Mann-Whitney U for non-distributed continuous variables, and Chi” test for categorical variables. For categorical variables with less than five observations Fisher’s exact test was used. *p < 0.05, **p < 0.001 *vs.* controls. Preterm delivery (n = 12) and hypertension (n = 7) which is not included in these groups, are excluded from the control group. ^a^before pregnancy, ^b^missing one value (Ctrl = 1), ^c^missing eight values (Ctrl = 3, GDM = 3, PE = 2) in RNAseq and 100 values in qPCR, ^d^initiated by oxytocin or prostaglandin, missing five values (Ctrl = 2, GDM = 3) in RNAseq and 50 values in qPCR.

**Table 2:** Predictors of *CXCL14* and *ANGPT2* expression in the placenta in all patients (RT-qPCR, n = 475).

Variables	*ANGPT2*				*CXCL14*			
	Univariate		Multivariable		Univariate		Multivariable	
	r	p	β	p	r	p	β	p
Age	0.03	0.552			0.06	0.182		
BMI v4	−0.01	0.765			0.09	0.063		
Previous smoke	0.06	0.188			0.03	0.587		
Parity^1^	0.19	<0.001	0.17	<0.001	0.16	<0.001	0.12	0.012
Systolic BP v4	0.02	0.695			−0.01	0.793		
Diastolic BP v4	−0.05	0.286			−0.03	0.571		
Gestational age	−0.24	<0.001	−0.22	<0.001	−0.18	<0.001	−0.24	<0.001
Birth weight	−0.09	0.042			0.10	0.031	0.15	0.004
Delivery mode^2^	0.05	0.325			0.03	0.486		
Spinal anesthesia	0.06	0.227			0.06	0.217		
Epidural anesthesia	−0.05	0.270			−0.13	0.005		
Offspring sex	−0.26	<0.001	−0.22	<0.001	0.03	0.528		
Placental weight	−0.01	0.919			0.08	0.077		
GDM (WHO criteria)	−0.03	0.610			0.07	0.139		
GDM (IADPSG criteria)	0.04	0.382			0.08	0.062		
Pre-eclampsia	−0.01	0.755			−0.01	0.943		
R square			0.15				0.08	

^1^nulliparous/multiparous.

^2^vaginal/cesarean section. Duration of labor, and induction of labor was not included due to missing data, 100 and 50, respectively.
