# Supplementary material for: Gene expression in term placentas is regulated more by spinal or epidural anesthesia than by late-onset preeclampsia or gestational diabetes mellitus
Source: Sci Rep. 2016 Jul 11;6:29715. doi: 10.1038/srep29715 (PMC4942618; doi:10.1038/srep29715)

Supplemental data

Gene expression in term placentas is regulated more by spinal or epidural anesthesia than by late-onset preeclampsia or gestational diabetes mellitus

Tove Lekva, Robert Lyle, Marie Cecilie Paasche Roland, Camilla Friis, Diana W. Bianchi, Iris Z. Jaffe, Errol R. Norwitz, Jens Bollerslev, Tore Henriksen, Thor Ueland

**Supplemental Table 1.** Maternal, fetal and delivery specific variables in women

with normal pregnancies (Ctrl), gestational diabetes (GDM, WHO) and pre-eclampsia

RT-qPCR (n=475) cohorts.

|  | **RNAseq** | | |
| --- | --- | --- | --- |
| **Characteristics** | **Ctrl (394)** | **GDM (62)** | **PE (19)** |
| **Maternal** |  |  |  |
| Age (years) | 31.4±3.9 | 31.8±4.9 | 30.4±3.8 |
| BMI visit 1 | 24.0 (21.8, 26.1) | 25.7 (23.1, 28.0)** | 28.6 (24.5, 31.4)** |
| BMI visit 4 | 27.6 (25.3, 30.1) | 28.7 (25.6, 31.4) | 32.6 (27.3, 35.4)** |
| Smokers^a^ n (%) | 85 (22) | 16 (27) | 3 (16) |
| Primipara n (%) | 202 (52) | 30 (50) | 13 (68) |
| SBP visit 1 | 110 (100, 120) | 110 (106, 120)* | 120 (110, 120)** |
| SBP visit 4 | 110 (100, 120) | 110 (106, 120) | 130 (120, 138)** |
| DBP visit 1 | 65 (60, 70) | 70 (61, 70)* | 70 (65, 80)* |
| DBP visit 4 | 70 (65, 80) | 70 (69, 75) | 83 (76, 90)** |
| **Delivery specific** |  |  |  |
| Anesthesia (spinal, epidural,  pudendal, general), n (%) | 68,162,12,5  (17,41,3,1) | 16,22,5,0  (26,36,8,0) | 4,8,1,0  (21,42,5,0) |
| C-section, n (%) | 67 (17) | 15 (24) | 5 (26) |
| Duration of labor (hours)^b^ | 6.0 (4.0, 10.8) | 6.0 (4.0, 11.0) | 3.8 (2.0, 7.0)* |
| Initiated labor, n (%)^c^ | 71 (20) | 15 (29) | 11 (64)** |
| **Fetal** |  |  |  |
| Gestational age (weeks) | 40.3 (39.4, 41.0) | 39.9 (38.9, 40.9) | 39.7 (38.6, 40.3)* |
| Birth weight (g) | 3659±489 | 3715±485 | 3698±589 |
| Girls, (%) | 215 (55) | 33 (53) | 11 (58) |
| Placenta weight (g) | 700 (620, 815) | 710 (600, 870) | 700 (610, 760) |

Data given as mean±SD when normal distributed and median (25^th^, 75^th^) when skewed distributed.

Comparison between placentas from women with GDM and controls, and PE and controls, were

performed using t-test for normal distributed variables, Mann-Whitney U for non-distributed

continuous variables, and Chi” test for categorical variables. *p<0.05 , **p<0.001 *vs.* controls. Preterm delivery (n=12) and hypertension (n=7) which is not included in these groups, are excluded from the control group

^a^ before pregnancy,^b^missing eight values (Ctrl=3, GDM=3, PE=2) in RNAseq and 100 values in qPCR, ^c^ initiated by oxytocin or prostaglandin, missing five values (Ctrl=2, GDM=3) in RNAseq and 50 values in qPCR.

**Supplemental Table 2.** Differences in some maternal, fetal and delivery specific variables in women with or without epidural or spinal anesthesia

|  |  | **Epidural** | |  |  | **Spinal** | |  |
| --- | --- | --- | --- | --- | --- | --- | --- | --- |
| **Characteristics** | **Missing, n** | **yes (n=7)** | **no (n=12)** | **p-value** | **Missing, n** | **yes (n=9)** | **no (n=12)** | **p-value** |
| Age (years) | 0 | 31±4 | 32±4 | 0.78 | 0 | 30±3 | 32±4 | 0.21 |
| BMI visit 4 (kg/m^2^) | 1 (ctrl) | 30.8±3.5 | 27.5±5.0 | 0.16 | 2 | 29.9±5.4 | 27.5±5.0 | 0.33 |
| Smokers^a^ n (%) | 0 | 0 (0) | 2 (16.7) | 0.39 | 0 | 2 (22.2) | 2 (16.7) | 0.59 |
| Primipara n (%) | 1 (ctrl) | 5 (71.4) | 6 (54.5) | 0.34 | 1 (case) | 3 (37.5) | 6 (50.0) | 0.47 |
| SBP visit 4 (mmHg) | 1 (ctrl) | 122±17 | 119±10 | 0.64 | 2 | 130±19 | 119±10 | 0.12 |
| DBP visit 4 (mmHg) | 1 (ctrl) | 80±17 | 76±8 | 0.49 | 2 | 78±13 | 76±8 | 0.65 |
| Hypertension, n (%) | 0 | 2 (28.6) | 3 (25.0) | 0.63 | 0 | 1 (11.1) | 3 (25.0) | 0.41 |
| Gestational age (yrs) | 0 | 40.1±0.7 | 39.6±1.2 | 0.30 | 0 | 39.6±0.7 | 39.6±1.2 | 0.98 |
| GDM (WHO/IADPSG) | 0 | 2(28.6)/2 (28.6) | 3(25.0)/4 (33.3) | 0.42/0.58 | 0 | 1(11.1)/2(22.2) | 3(25)/4(33.3) | 0.34/0.37 |
| PE | 0 | 4 (57.1) | 4 (33.3) | 0.30 | 0 | 2 (22.2) | 4 (33.3) | 0.48 |
| Cesarean delivery | 0 |  |  |  | 0 | 7 (77.8) | 0 (0) | <0.001 |
| Induced labor^b^ | 0 | 2 (28.5) | 3 (25.0) | 0.63 | 4 (cases) | 2 (40.0) | 3 (25.0) | 0.47 |
| Umbilical cord venous base excess | 4 (ctrl) | -6.9±2.4 | -6.4±2.7 | 0.67 | 5 (4 ctrl, 1 case) | -2.4±2.3 | -6.4±2.7 | 0.006 |
| Blood loss > 1000 mL | 9 (6 ctrl, 3 cases) | 1 (25.0) | 1 (16.7) | 0.67 | 10 (6 ctrl, 4 cases) | 0 (0) | 1 (16.7) | 0.55 |
| Placenta weight | 0 | 685±121 | 718±160 | 0.64 | 0 | 770±156 | 718±160 | 0.47 |

^a^ previous, ^b^ initiated by oxytocin or prostaglandin.

Supplemental Table 3A. List of the 70 DEGs from the placenta investigating maternal variables sorted on FDR and unadjusted p-values.

Parity

| **symbol** | **log2FC** | **p-value** | **p-adjust.** | **Ensembl** |
| --- | --- | --- | --- | --- |
| FAM107A | -0.93 | 4.34E-08 | 0.0007 | ENSG00000168309 |
| PRX | -0.74 | 6.67E-08 | 0.0007 | ENSG00000105227 |
| PTX3 | -0.83 | 1.48E-06 | 0.0074 | ENSG00000163661 |
| CCDC141 | -0.75 | 1.51E-06 | 0.0074 | ENSG00000163492 |
| HYAL2 | -0.69 | 2.09E-06 | 0.0082 | ENSG00000068001 |
| ZBTB16 | -0.79 | 3.09E-06 | 0.0101 | ENSG00000109906 |
| PDE9A | -0.72 | 4.69E-06 | 0.0131 | ENSG00000160191 |
| MCF2L | -0.78 | 5.60E-06 | 0.0137 | ENSG00000126217 |
| CX3CR1 | 0.66 | 8.24E-06 | 0.0179 | ENSG00000168329 |
| NA | -0.74 | 1.48E-05 | 0.0265 | ENSG00000248636 |
| PITPNC1 | -0.63 | 1.49E-05 | 0.0265 | ENSG00000154217 |
| LOC102724190 | -0.75 | 1.62E-05 | 0.0265 | ENSG00000258819 |
| APOLD1 | -0.75 | 1.77E-05 | 0.0266 | ENSG00000178878 |
| PDK4 | -0.58 | 2.89E-05 | 0.0404 | ENSG00000004799 |
| SDPR | -0.65 | 6.77E-05 | 0.0883 | ENSG00000168497 |
| TSC22D3 | -0.64 | 7.89E-05 | 0.0964 | ENSG00000157514 |
| WNT7B | 0.64 | 0.0001 | 0.1137 | ENSG00000188064 |
| NA | -0.66 | 0.0001 | 0.1137 | ENSG00000272321 |
| SNHG20 | -0.39 | 0.0001 | 0.1137 | ENSG00000234912 |
| CCDC134 | 0.52 | 0.0001 | 0.1137 | ENSG00000100147 |
| IL18R1 | -0.64 | 0.0001 | 0.1137 | ENSG00000115604 |
| DLL1 | -0.66 | 0.0001 | 0.1200 | ENSG00000198719 |
| IL4R | -0.33 | 0.0001 | 0.1200 | ENSG00000077238 |
| KLF9 | -0.60 | 0.0002 | 0.1344 | ENSG00000119138 |
| ZNF469 | 0.59 | 0.0002 | 0.1455 | ENSG00000225614 |
| EDNRB | -0.50 | 0.0002 | 0.1466 | ENSG00000136160 |
| PCK1 | -0.59 | 0.0002 | 0.1545 | ENSG00000124253 |
| NA | -0.59 | 0.0002 | 0.1545 | ENSG00000270956 |
| LOC102723927 | -0.63 | 0.0002 | 0.1545 | ENSG00000237940 |
| SCGN | -0.64 | 0.0002 | 0.1568 | ENSG00000079689 |
| LMO2 | 0.52 | 0.0003 | 0.1621 | ENSG00000135363 |
| FKBP5 | -0.61 | 0.0003 | 0.1711 | ENSG00000096060 |
| ITGA10 | -0.57 | 0.0003 | 0.1711 | ENSG00000143127 |
| PRCD | -0.59 | 0.0003 | 0.1744 | ENSG00000214140 |
| DGKG | -0.59 | 0.0003 | 0.1751 | ENSG00000058866 |
| DOC2B | -0.53 | 0.0003 | 0.1796 | ENSG00000272636 |
| PPEF1 | -0.62 | 0.0004 | 0.1808 | ENSG00000086717 |
| EMP1 | -0.53 | 0.0004 | 0.1808 | ENSG00000134531 |
| NA | -0.60 | 0.0005 | 0.2230 | ENSG00000279447 |
| ST6GALNAC1 | -0.58 | 0.0005 | 0.2230 | ENSG00000070526 |
| CDKL1 | -0.49 | 0.0005 | 0.2230 | ENSG00000100490 |
| NA | 0.42 | 0.0005 | 0.2485 | ENSG00000169246 |
| KCNS2 | -0.59 | 0.0006 | 0.2753 | ENSG00000156486 |
| IL1RL1 | -0.60 | 0.0006 | 0.2757 | ENSG00000115602 |
| SLA | -0.51 | 0.0007 | 0.3076 | ENSG00000155926 |
| MAP3K6 | -0.51 | 0.0007 | 0.3076 | ENSG00000142733 |
| PIK3R3 | -0.52 | 0.0008 | 0.3076 | ENSG00000117461 |
| TMEM233 | -0.59 | 0.0008 | 0.3076 | ENSG00000224982 |
| PDE8B | -0.52 | 0.0009 | 0.3076 | ENSG00000113231 |
| DKK2 | 0.58 | 0.0009 | 0.3076 | ENSG00000155011 |
| PTPN20 | -0.58 | 0.0009 | 0.3076 | ENSG00000204179 |
| F2RL3 | -0.54 | 0.0009 | 0.3076 | ENSG00000127533 |
| CASC10 | 0.37 | 0.0009 | 0.3076 | ENSG00000204682 |
| PRIM2 | 0.26 | 0.0009 | 0.3076 | ENSG00000146143 |
| RAPGEF3 | -0.43 | 0.0009 | 0.3076 | ENSG00000079337 |
| NFYB | -0.32 | 0.0009 | 0.3076 | ENSG00000120837 |
| ADIPOR2 | -0.25 | 0.0009 | 0.3076 | ENSG00000006831 |
| NT5C3B | -0.36 | 0.0009 | 0.3076 | ENSG00000141698 |
| LOC102725182 | -0.52 | 0.0009 | 0.3076 | ENSG00000250138 |
| ARRDC2 | -0.51 | 0.0010 | 0.3076 | ENSG00000105643 |
| ACSL1 | -0.51 | 0.0010 | 0.3076 | ENSG00000151726 |
| KLF15 | -0.57 | 0.0010 | 0.3076 | ENSG00000163884 |
| IL2RA | -0.52 | 0.0010 | 0.3076 | ENSG00000134460 |
| IRAK3 | -0.47 | 0.0010 | 0.3141 | ENSG00000090376 |
| LOC101928612 | -0.57 | 0.0010 | 0.3141 | ENSG00000228401 |
| TACR3 | -0.57 | 0.0011 | 0.3206 | ENSG00000169836 |
| MT1M | -0.56 | 0.0011 | 0.3315 | ENSG00000205364 |
| JADE1 | -0.40 | 0.0012 | 0.3315 | ENSG00000077684 |
| GGT1 | 0.44 | 0.0012 | 0.3315 | ENSG00000100031 |
| CA4 | -0.56 | 0.0012 | 0.3322 | ENSG00000167434 |

BMI

| **symbol** | **log2FC** | **p-value** | **p-adjust.** | **Ensembl** |
| --- | --- | --- | --- | --- |
| CFD | 1.19 | 4.51E-06 | 0.0636 | ENSG00000197766 |
| HSPA1A | 1.12 | 1.14E-05 | 0.0805 | ENSG00000204389 |
| SNCA | 1.00 | 2.56E-05 | 0.0949 | ENSG00000145335 |
| GYPA | 1.25 | 3.12E-05 | 0.0949 | ENSG00000170180 |
| PPM1M | 0.34 | 3.36E-05 | 0.0949 | ENSG00000164088 |
| RBM38 | 0.79 | 4.77E-05 | 0.0961 | ENSG00000132819 |
| ZSCAN12P1 | -0.79 | 4.77E-05 | 0.0961 | ENSG00000219891 |
| CYP4B1 | 1.27 | 6.02E-05 | 0.0961 | ENSG00000142973 |
| GMPR | 0.53 | 6.13E-05 | 0.0961 | ENSG00000137198 |
| IQCG | 0.97 | 9.62E-05 | 0.1257 | ENSG00000114473 |
| ANK1 | 0.67 | 9.79E-05 | 0.1257 | ENSG00000029534 |
| HSPA1B | 1.14 | 0.000131 | 0.1303 | ENSG00000204388 |
| AHSP | 1.11 | 0.000132 | 0.1303 | ENSG00000169877 |
| TMEM70 | 0.37 | 0.000136 | 0.1303 | ENSG00000175606 |
| HBG2 | 1.08 | 0.000138 | 0.1303 | ENSG00000196565 |
| IER5L | 0.97 | 0.000162 | 0.1432 | ENSG00000188483 |
| NINJ1 | 0.30 | 0.000189 | 0.1567 | ENSG00000131669 |
| TMCC2 | 0.91 | 0.000232 | 0.1735 | ENSG00000133069 |
| SLC25A37 | 0.54 | 0.000254 | 0.1735 | ENSG00000147454 |
| APOE | 0.63 | 0.000254 | 0.1735 | ENSG00000130203 |
| ARL4D | 0.56 | 0.000273 | 0.1735 | ENSG00000175906 |
| ALAS2 | 1.02 | 0.000308 | 0.1735 | ENSG00000158578 |
| IFIT1B | 1.03 | 0.000333 | 0.1735 | ENSG00000204010 |
| GADD45A | 0.67 | 0.000334 | 0.1735 | ENSG00000116717 |
| LSS | -0.66 | 0.000337 | 0.1735 | ENSG00000160285 |
| FAM46C | 0.82 | 0.000341 | 0.1735 | ENSG00000183508 |
| C1orf50 | 0.33 | 0.000342 | 0.1735 | ENSG00000164008 |
| HBG1 | 1.06 | 0.000344 | 0.1735 | ENSG00000213934 |
| MDK | 0.53 | 0.000388 | 0.1890 | ENSG00000110492 |
| GYPB | 1.03 | 0.000451 | 0.2079 | ENSG00000250361 |
| ZNF658 | -0.55 | 0.000457 | 0.2079 | ENSG00000274349 |
| TCN2 | 0.72 | 0.000485 | 0.2079 | ENSG00000185339 |
| SLC25A39 | 0.43 | 0.000486 | 0.2079 | ENSG00000013306 |
| HBM | 1.01 | 0.000561 | 0.2129 | ENSG00000206177 |
| FCGRT | 0.46 | 0.000561 | 0.2129 | ENSG00000104870 |
| SLC4A1 | 0.93 | 0.000563 | 0.2129 | ENSG00000004939 |
| NA | 0.58 | 0.000589 | 0.2129 | ENSG00000254615 |
| APOC1 | 0.75 | 0.000597 | 0.2129 | ENSG00000130208 |
| FERMT3 | 0.34 | 0.000601 | 0.2129 | ENSG00000149781 |
| PFN1 | 0.36 | 0.000607 | 0.2129 | ENSG00000108518 |
| PTGDS | 0.70 | 0.000618 | 0.2129 | ENSG00000107317 |
| DMTN | 0.77 | 0.000637 | 0.2141 | ENSG00000158856 |
| UNC93B1 | 0.48 | 0.000689 | 0.2262 | ENSG00000110057 |
| HBA1 | 0.99 | 0.000726 | 0.2328 | ENSG00000206172 |
| KCNQ1 | 0.40 | 0.000749 | 0.2350 | ENSG00000053918 |
| CYBA | 0.46 | 0.00079 | 0.2376 | ENSG00000051523 |
| ADGRL3 | -0.92 | 0.0008 | 0.2376 | ENSG00000150471 |
| NA | 0.97 | 0.000808 | 0.2376 | ENSG00000279346 |
| RRAS | 0.44 | 0.000832 | 0.2396 | ENSG00000126458 |
| RAB20 | 0.51 | 0.000858 | 0.2424 | ENSG00000139832 |
| HBA2 | 0.96 | 0.000916 | 0.2513 | ENSG00000188536 |
| CXCL14 | 0.70 | 0.000926 | 0.2513 | ENSG00000145824 |
| TMOD1 | 0.78 | 0.000969 | 0.2518 | ENSG00000136842 |
| RAB3IL1 | 0.45 | 0.000983 | 0.2518 | ENSG00000167994 |
| PDSS1 | 0.41 | 0.000984 | 0.2518 | ENSG00000148459 |
| TWF2 | 0.40 | 0.001007 | 0.2518 | ENSG00000247596 |
| GNA15 | 0.49 | 0.001016 | 0.2518 | ENSG00000060558 |
| PRSS23 | 0.64 | 0.001051 | 0.2521 | ENSG00000150687 |
| ACTB | 0.48 | 0.001054 | 0.2521 | ENSG00000075624 |
| SCARF2 | 0.50 | 0.001096 | 0.2578 | ENSG00000244486 |
| BTK | 0.36 | 0.001125 | 0.2603 | ENSG00000010671 |
| RNF130 | 0.30 | 0.001149 | 0.2616 | ENSG00000113269 |
| RARRES2 | 0.57 | 0.001191 | 0.2670 | ENSG00000106538 |
| R3HDM4 | 0.43 | 0.001215 | 0.2680 | ENSG00000198858 |
| OSBP2 | 0.62 | 0.00126 | 0.2721 | ENSG00000184792 |
| XK | 0.61 | 0.001277 | 0.2721 | ENSG00000047597 |
| MZT2B | 0.59 | 0.001316 | 0.2721 | ENSG00000152082 |
| MOSPD3 | 0.48 | 0.001339 | 0.2721 | ENSG00000106330 |
| GYPC | 0.59 | 0.001394 | 0.2721 | ENSG00000136732 |
| SH3BGRL3 | 0.43 | 0.001401 | 0.2721 | ENSG00000142669 |

Supplemental Table 3B. List of the 70 DEGs from the placenta investigating delivery specific variables sorted on FDR and unadjusted p-values.

**Epidural anesthesia**

| **symbol** | **log2FC** | **p-value** | **p-adjust.** | **Ensembl** |
| --- | --- | --- | --- | --- |
| HSPA1A | 1.38 | 2.27E-10 | 4.02E-06 | ENSG00000204389 |
| CHORDC1 | 0.69 | 2.47E-09 | 2.19E-05 | ENSG00000110172 |
| HSPA1B | 1.16 | 1.13E-07 | 0.0005 | ENSG00000204388 |
| DNAJA4 | 1.01 | 1.19E-07 | 0.0005 | ENSG00000140403 |
| HSPA4L | 0.97 | 1.61E-07 | 0.0006 | ENSG00000164070 |
| HSPH1 | 0.88 | 9.48E-07 | 0.0028 | ENSG00000120694 |
| DNAJB1 | 0.91 | 1.11E-05 | 0.0282 | ENSG00000132002 |
| JMJD6 | 0.50 | 1.76E-05 | 0.0390 | ENSG00000070495 |
| ERICH2 | 0.92 | 2.70E-05 | 0.0492 | ENSG00000204334 |
| NA | -0.86 | 2.77E-05 | 0.0492 | ENSG00000277350 |
| HIST1H4H | 0.89 | 3.80E-05 | 0.0612 | ENSG00000158406 |
| RSRP1 | 0.46 | 6.32E-05 | 0.0763 | ENSG00000117616 |
| LOC100996425 | 0.87 | 6.65E-05 | 0.0763 | ENSG00000260896 |
| KRT24 | -0.66 | 7.05E-05 | 0.0763 | ENSG00000167916 |
| KRT17 | -0.85 | 7.17E-05 | 0.0763 | ENSG00000128422 |
| C1orf21 | 0.48 | 7.62E-05 | 0.0763 | ENSG00000116667 |
| HIST1H3D | 0.86 | 8.41E-05 | 0.0763 | ENSG00000197409 |
| UPK1B | -0.83 | 8.60E-05 | 0.0763 | ENSG00000114638 |
| BPI | 0.83 | 8.65E-05 | 0.0763 | ENSG00000101425 |
| HSPE1 | 0.68 | 8.78E-05 | 0.0763 | ENSG00000115541 |
| PADI2 | 0.66 | 9.74E-05 | 0.0763 | ENSG00000117115 |
| CACNG4 | -0.81 | 9.77E-05 | 0.0763 | ENSG00000075461 |
| KRT6A | -0.63 | 9.90E-05 | 0.0763 | ENSG00000205420 |
| NA | -0.78 | 0.0001 | 0.0786 | ENSG00000279806 |
| NA | -0.84 | 0.0001 | 0.0786 | ENSG00000236304 |
| HSP90AA1 | 0.65 | 0.0001 | 0.0802 | ENSG00000080824 |
| NA | -0.77 | 0.0001 | 0.0865 | ENSG00000281415 |
| HSPD1 | 0.47 | 0.0001 | 0.0921 | ENSG00000144381 |
| PARD6G-AS1 | 0.80 | 0.0002 | 0.0929 | ENSG00000267270 |
| CSRP2BP | -0.54 | 0.0002 | 0.0948 | ENSG00000149474 |
| THAP2 | 0.53 | 0.0002 | 0.0967 | ENSG00000173451 |
| MPO | 0.78 | 0.0002 | 0.0967 | ENSG00000005381 |
| CHRM5 | -0.81 | 0.0002 | 0.0967 | ENSG00000184984 |
| AOC1 | -0.81 | 0.0002 | 0.1012 | ENSG00000002726 |
| ZNF114 | 0.80 | 0.0002 | 0.1088 | ENSG00000178150 |
| HIST1H2AE | 0.80 | 0.0002 | 0.1088 | ENSG00000277075 |
| LANCL2 | -0.32 | 0.0002 | 0.1090 | ENSG00000132434 |
| SLC1A6 | -0.73 | 0.0002 | 0.1096 | ENSG00000105143 |
| REPS2 | -0.67 | 0.0003 | 0.1157 | ENSG00000169891 |
| C3orf18 | -0.46 | 0.0003 | 0.1172 | ENSG00000088543 |
| HIST1H4E | 0.79 | 0.0003 | 0.1318 | ENSG00000276966 |
| GKN1 | -0.72 | 0.0003 | 0.1318 | ENSG00000169605 |
| LOC101928707 | -0.78 | 0.0003 | 0.1342 | ENSG00000260219 |
| KDM8 | -0.49 | 0.0003 | 0.1342 | ENSG00000155666 |
| CEACAM8 | 0.77 | 0.0003 | 0.1342 | ENSG00000124469 |
| NA | 0.49 | 0.0004 | 0.1458 | ENSG00000258472 |
| NA | 0.75 | 0.0004 | 0.1564 | ENSG00000262133 |
| FAM43B | -0.74 | 0.0005 | 0.1842 | ENSG00000183114 |
| FN1 | -0.76 | 0.0005 | 0.1842 | ENSG00000115414 |
| HSPA8 | 0.50 | 0.0005 | 0.1842 | ENSG00000109971 |
| NA | 0.69 | 0.0005 | 0.1842 | ENSG00000279821 |
| TIGD6 | -0.40 | 0.0006 | 0.1842 | ENSG00000164296 |
| IL17RE | -0.75 | 0.0006 | 0.1842 | ENSG00000163701 |
| GNA13 | 0.25 | 0.0006 | 0.1842 | ENSG00000120063 |
| SFN | -0.71 | 0.0006 | 0.1856 | ENSG00000175793 |
| NUAK1 | -0.65 | 0.0006 | 0.1863 | ENSG00000074590 |
| DNAJA1 | 0.44 | 0.0006 | 0.1863 | ENSG00000086061 |
| NOP14-AS1 | -0.44 | 0.0006 | 0.1863 | ENSG00000249673 |
| TPM3P9 | 0.53 | 0.0006 | 0.1911 | ENSG00000241015 |
| SCARNA7 | -0.74 | 0.0007 | 0.1933 | ENSG00000238741 |
| HIST2H2BF | 0.66 | 0.0007 | 0.1986 | ENSG00000203814 |
| KCNJ16 | -0.74 | 0.0007 | 0.1986 | ENSG00000153822 |
| EGFR-AS1 | -0.73 | 0.0008 | 0.2107 | ENSG00000224057 |
| PPID | 0.31 | 0.0008 | 0.2107 | ENSG00000171497 |
| CSNK1E | 0.42 | 0.0008 | 0.2107 | ENSG00000213923 |
| AMZ1 | -0.73 | 0.0008 | 0.2107 | ENSG00000174945 |
| MCRS1 | -0.21 | 0.0008 | 0.2107 | ENSG00000187778 |
| NA | -0.65 | 0.0008 | 0.2107 | ENSG00000258897 |
| MS4A3 | 0.70 | 0.0008 | 0.2107 | ENSG00000149516 |
| TNFRSF10B | 0.47 | 0.0008 | 0.2107 | ENSG00000120889 |

**Spinal anesthesia**

| **symbol** | **log2FC** | **p-value** | **p-adjust.** | **Ensembl** |
| --- | --- | --- | --- | --- |
| NA | 1.00 | 1.20E-06 | 0.0128 | ENSG00000211893 |
| SFRP1 | -0.98 | 6.59E-07 | 0.0128 | ENSG00000104332 |
| CXCL9 | 0.93 | 2.38E-06 | 0.0170 | ENSG00000138755 |
| LOC102724332 | 0.73 | 4.27E-06 | 0.0228 | ENSG00000211592 |
| MISP | -0.93 | 8.03E-06 | 0.0338 | ENSG00000099812 |
| LAMA3 | -0.94 | 9.51E-06 | 0.0338 | ENSG00000053747 |
| KCNJ16 | -0.94 | 1.19E-05 | 0.0363 | ENSG00000153822 |
| KRT17 | -0.91 | 1.72E-05 | 0.0401 | ENSG00000128422 |
| KRT6A | -0.71 | 1.74E-05 | 0.0401 | ENSG00000205420 |
| PNPLA3 | -0.81 | 1.88E-05 | 0.0401 | ENSG00000100344 |
| CXCL10 | 0.89 | 2.26E-05 | 0.0425 | ENSG00000169245 |
| MTERF1 | 0.35 | 2.58E-05 | 0.0425 | ENSG00000127989 |
| KRT24 | -0.69 | 2.59E-05 | 0.0425 | ENSG00000167916 |
| NA | -0.82 | 3.13E-05 | 0.0478 | ENSG00000258897 |
| TRIM64B | -0.80 | 3.66E-05 | 0.0520 | ENSG00000189253 |
| FAM83A | -0.81 | 4.42E-05 | 0.0589 | ENSG00000147689 |
| SPRR2G | -0.77 | 4.92E-05 | 0.0617 | ENSG00000159516 |
| NA | 0.65 | 5.51E-05 | 0.0653 | ENSG00000241351 |
| NA | 0.65 | 6.01E-05 | 0.0676 | ENSG00000211677 |
| BAIAP2L1 | -0.51 | 6.57E-05 | 0.0701 | ENSG00000006453 |
| FAM46B | -0.79 | 8.10E-05 | 0.0823 | ENSG00000158246 |
| PRKAR1B | -0.65 | 0.0001 | 0.0933 | ENSG00000188191 |
| FGF12 | 0.82 | 0.0001 | 0.0933 | ENSG00000114279 |
| NA | -0.79 | 0.0001 | 0.0933 | ENSG00000277350 |
| MYOZ1 | -0.82 | 0.0001 | 0.0933 | ENSG00000177791 |
| SLC1A6 | -0.76 | 0.0001 | 0.0933 | ENSG00000105143 |
| UPK1B | -0.80 | 0.0001 | 0.0933 | ENSG00000114638 |
| IGJ | 0.70 | 0.0001 | 0.0933 | ENSG00000132465 |
| GJB3 | -0.81 | 0.0001 | 0.0933 | ENSG00000188910 |
| SFN | -0.79 | 0.0001 | 0.0933 | ENSG00000175793 |
| MARCH1 | 0.68 | 0.0001 | 0.0933 | ENSG00000145416 |
| AOC1 | -0.81 | 0.0001 | 0.0933 | ENSG00000002726 |
| EGFR-AS1 | -0.81 | 0.0001 | 0.0933 | ENSG00000224057 |
| NUAK2 | -0.73 | 0.0001 | 0.0933 | ENSG00000163545 |
| PCSK6 | -0.68 | 0.0002 | 0.0985 | ENSG00000140479 |
| FN1 | -0.79 | 0.0002 | 0.1168 | ENSG00000115414 |
| AMZ1 | -0.79 | 0.0002 | 0.1229 | ENSG00000174945 |
| CDH16 | -0.69 | 0.0002 | 0.1306 | ENSG00000166589 |
| PPP4R4 | -0.78 | 0.0003 | 0.1362 | ENSG00000119698 |
| LIAS | 0.37 | 0.0003 | 0.1362 | ENSG00000121897 |
| NOL7 | 0.28 | 0.0003 | 0.1388 | ENSG00000225921 |
| ZBED2 | -0.68 | 0.0003 | 0.1424 | ENSG00000177494 |
| SLC25A25 | -0.46 | 0.0003 | 0.1556 | ENSG00000148339 |
| NA | -0.77 | 0.0003 | 0.1584 | ENSG00000236304 |
| HS6ST1 | -0.49 | 0.0003 | 0.1601 | ENSG00000136720 |
| RUSC2 | -0.54 | 0.0004 | 0.1605 | ENSG00000198853 |
| SRF | -0.53 | 0.0004 | 0.1605 | ENSG00000112658 |
| F7 | -0.74 | 0.0004 | 0.1605 | ENSG00000057593 |
| XCR1 | -0.75 | 0.0004 | 0.1702 | ENSG00000173578 |
| HLA-G | -0.72 | 0.0004 | 0.1831 | ENSG00000204632 |
| ENTPD2 | -0.66 | 0.0005 | 0.1898 | ENSG00000054179 |
| KRT5 | -0.61 | 0.0005 | 0.1898 | ENSG00000186081 |
| ZNF510 | 0.38 | 0.0005 | 0.1898 | ENSG00000081386 |
| NA | -0.74 | 0.0005 | 0.1908 | ENSG00000261599 |
| KCNJ2 | -0.58 | 0.0005 | 0.1908 | ENSG00000123700 |
| AIFM3 | -0.72 | 0.0005 | 0.1908 | ENSG00000183773 |
| EGLN3 | -0.74 | 0.0005 | 0.1908 | ENSG00000129521 |
| ANKRD36BP2 | 0.74 | 0.0005 | 0.1945 | ENSG00000230006 |
| LINC00998 | 0.46 | 0.0005 | 0.1956 | ENSG00000214194 |
| YAE1D1 | 0.35 | 0.0006 | 0.1962 | ENSG00000241127 |
| NA | 0.74 | 0.0006 | 0.1964 | ENSG00000211896 |
| LHX5 | -0.58 | 0.0006 | 0.1996 | ENSG00000089116 |
| ZDHHC9 | -0.49 | 0.0006 | 0.2168 | ENSG00000188706 |
| LGI2 | 0.51 | 0.0007 | 0.2189 | ENSG00000153012 |
| SLC12A3 | -0.72 | 0.0007 | 0.2253 | ENSG00000070915 |
| EMB | 0.48 | 0.0007 | 0.2253 | ENSG00000170571 |
| MRPL39 | 0.27 | 0.0007 | 0.2253 | ENSG00000154719 |
| FBLIM1 | -0.49 | 0.0007 | 0.2253 | ENSG00000162458 |
| GDPD2 | -0.69 | 0.0007 | 0.2255 | ENSG00000130055 |
| FSTL3 | -0.72 | 0.0008 | 0.2283 | ENSG00000070404 |

Labor duration

| **symbol** | **log2FC** | **p-value** | **p-adjust.** | **Ensembl** |
| --- | --- | --- | --- | --- |
| YBX3 | 0.32 | 2.95E-06 | 0.0478 | ENSG00000060138 |
| HIST1H4D | 1.01 | 4.13E-06 | 0.0478 | ENSG00000277157 |
| C1orf21 | 0.47 | 1.14E-05 | 0.0633 | ENSG00000116667 |
| HSPA1A | 0.95 | 1.37E-05 | 0.0633 | ENSG00000204389 |
| ARG1 | 0.98 | 9.65E-06 | 0.0633 | ENSG00000118520 |
| LOC100996425 | 0.93 | 2.01E-05 | 0.0776 | ENSG00000260896 |
| HIST1H4E | 0.90 | 2.97E-05 | 0.0983 | ENSG00000276966 |
| HPSE2 | -0.87 | 6.58E-05 | 0.1783 | ENSG00000172987 |
| S100A8 | 0.87 | 7.37E-05 | 0.1783 | ENSG00000143546 |
| NA | 0.66 | 8.00E-05 | 0.1783 | ENSG00000264207 |
| HK2 | 0.86 | 8.47E-05 | 0.1783 | ENSG00000159399 |
| IFNA1 | 0.74 | 0.0001 | 0.1943 | ENSG00000197919 |
| HIST1H4B | 0.81 | 0.0001 | 0.2112 | ENSG00000278705 |
| BHLHE40-AS1 | 0.71 | 0.0001 | 0.2175 | ENSG00000235831 |
| MS4A3 | 0.82 | 0.0002 | 0.2419 | ENSG00000149516 |
| HSPA1B | 0.83 | 0.0002 | 0.2419 | ENSG00000204388 |
| KIF26B | -0.67 | 0.0002 | 0.2475 | ENSG00000162849 |
| NA | 0.82 | 0.0002 | 0.2475 | ENSG00000250509 |
| PCAT6 | 0.70 | 0.0003 | 0.3321 | ENSG00000228288 |
| S100A12 | 0.80 | 0.0003 | 0.3516 | ENSG00000163221 |
| VPREB3 | 0.77 | 0.0003 | 0.3522 | ENSG00000128218 |
| NA | 0.66 | 0.0004 | 0.3522 | ENSG00000267222 |
| NA | 0.71 | 0.0004 | 0.3522 | ENSG00000277675 |
| ASB15 | -0.73 | 0.0004 | 0.3522 | ENSG00000146809 |
| NMRK1 | 0.41 | 0.0004 | 0.3522 | ENSG00000106733 |
| CYP2D7 | 0.68 | 0.0004 | 0.3522 | ENSG00000205702 |
| HIST1H4C | 0.59 | 0.0004 | 0.3522 | ENSG00000197061 |
| DBH | -0.77 | 0.0004 | 0.3522 | ENSG00000123454 |
| ZNF362 | -0.45 | 0.0004 | 0.3522 | ENSG00000160094 |
| PPP1R1C | 0.70 | 0.0005 | 0.3522 | ENSG00000150722 |
| NA | 0.73 | 0.0005 | 0.3957 | ENSG00000236663 |
| SCARNA21 | 0.67 | 0.0005 | 0.3957 | ENSG00000252835 |
| VCX | -0.75 | 0.0006 | 0.4042 | ENSG00000182583 |
| MPO | 0.74 | 0.0006 | 0.4165 | ENSG00000005381 |
| FGFR2 | -0.47 | 0.0007 | 0.4165 | ENSG00000066468 |
| NA | 0.72 | 0.0007 | 0.4165 | ENSG00000236358 |
| FREM2 | -0.57 | 0.0007 | 0.4165 | ENSG00000150893 |
| LDHA | 0.55 | 0.0007 | 0.4165 | ENSG00000134333 |
| ZNRD1 | 0.32 | 0.0007 | 0.4165 | ENSG00000066379 |
| DNAJB1 | 0.70 | 0.0008 | 0.4165 | ENSG00000132002 |
| SERPINB7 | 0.73 | 0.0008 | 0.4165 | ENSG00000166396 |
| FBXO24 | -0.65 | 0.0008 | 0.4165 | ENSG00000106336 |
| NA | 0.74 | 0.0008 | 0.4165 | ENSG00000263081 |
| VEGFA | 0.68 | 0.0008 | 0.4165 | ENSG00000112715 |
| HSPH1 | 0.62 | 0.0009 | 0.4165 | ENSG00000120694 |
| TIFA | 0.48 | 0.0009 | 0.4165 | ENSG00000145365 |
| RPS26 | 0.61 | 0.0009 | 0.4165 | ENSG00000197728 |
| PADI4 | 0.72 | 0.0009 | 0.4165 | ENSG00000159339 |
| NAAA | 0.40 | 0.0009 | 0.4165 | ENSG00000138744 |
| LOC101927809 | 0.69 | 0.0010 | 0.4165 | ENSG00000260552 |
| TMEM251 | 0.37 | 0.0010 | 0.4165 | ENSG00000153485 |
| TDO2 | -0.68 | 0.0010 | 0.4165 | ENSG00000151790 |
| FBXO2 | 0.72 | 0.0010 | 0.4165 | ENSG00000116661 |
| MGC16275 | -0.71 | 0.0010 | 0.4165 | ENSG00000246731 |
| SGK223 | -0.53 | 0.0010 | 0.4165 | ENSG00000275342 |
| PDK1 | 0.42 | 0.0010 | 0.4165 | ENSG00000152256 |
| LOC100507140 | 0.70 | 0.0010 | 0.4165 | ENSG00000237166 |
| FAM64A | -0.72 | 0.0010 | 0.4165 | ENSG00000129195 |
| NA | -0.54 | 0.0011 | 0.4165 | ENSG00000260461 |
| JMJD6 | 0.39 | 0.0011 | 0.4179 | ENSG00000070495 |
| NA | 0.66 | 0.0011 | 0.4257 | ENSG00000226318 |
| NA | 0.71 | 0.0012 | 0.4257 | ENSG00000224945 |
| KCNQ5 | -0.71 | 0.0012 | 0.4257 | ENSG00000185760 |
| WDR7 | -0.28 | 0.0012 | 0.4305 | ENSG00000091157 |
| EDN1 | -0.64 | 0.0012 | 0.4377 | ENSG00000078401 |
| CREM | 0.60 | 0.0012 | 0.4377 | ENSG00000095794 |
| MIR210HG | 0.71 | 0.0013 | 0.4461 | ENSG00000247095 |
| SEC14L4 | 0.71 | 0.0014 | 0.4629 | ENSG00000133488 |
| LOC102724279 | 0.61 | 0.0014 | 0.4647 | ENSG00000267453 |
| SPINK1 | 0.62 | 0.0014 | 0.4647 | ENSG00000164266 |

Vaginal/C-section

| **symbol** | | **log2FC** | | **pvalue** | | **padj** | | **Ensembl** | |
| --- | --- | --- | --- | --- | --- | --- | --- | --- | --- |
| NR4A3 | | -1.00 | | 6.55E-07 | | 0.0246 | | ENSG00000119508 | |
| FOSL2 | | -0.67 | | 4.95E-05 | | 0.9239 | | ENSG00000075426 | |
| NA | | -0.78 | | 8.75E-05 | | 0.9239 | | ENSG00000231487 | |
| HBEGF | | -0.75 | | 9.82E-05 | | 0.9239 | | ENSG00000113070 | |
| NA | 0.65 | | 0.0003 | | 0.9967 | | ENSG00000254614 | |  |
| NUAK2 | -0.66 | | 0.0003 | | 0.9967 | | ENSG00000163545 | |  |
| SLC16A6 | -0.63 | | 0.0004 | | 0.9967 | | ENSG00000108932 | |  |
| APOBEC3A | -0.70 | | 0.0004 | | 0.9967 | | ENSG00000128383 | |  |
| NA | 0.59 | | 0.0005 | | 0.9967 | | ENSG00000277463 | |  |
| PAX5 | -0.69 | | 0.0005 | | 0.9967 | | ENSG00000196092 | |  |
| PADI2 | -0.61 | | 0.0005 | | 0.9967 | | ENSG00000117115 | |  |
| EGR3 | -0.69 | | 0.0005 | | 0.9967 | | ENSG00000179388 | |  |
| PROK2 | -0.69 | | 0.0005 | | 0.9967 | | ENSG00000163421 | |  |
| LOC101928438 | -0.59 | | 0.0006 | | 0.9967 | | ENSG00000237461 | |  |
| NA | -0.68 | | 0.0006 | | 0.9967 | | ENSG00000255769 | |  |
| SCNN1G | -0.68 | | 0.0006 | | 0.9967 | | ENSG00000166828 | |  |
| CLEC18A | -0.68 | | 0.0007 | | 0.9967 | | ENSG00000140839 | |  |
| MYH14 | -0.68 | | 0.0007 | | 0.9967 | | ENSG00000105357 | |  |
| NA | -0.67 | | 0.0007 | | 0.9967 | | ENSG00000276570 | |  |
| F2RL3 | -0.65 | | 0.0008 | | 0.9967 | | ENSG00000127533 | |  |
| ALPL | -0.66 | | 0.0008 | | 0.9967 | | ENSG00000162551 | |  |
| MGST1 | -0.66 | | 0.0009 | | 0.9967 | | ENSG00000008394 | |  |
| MAP10 | 0.63 | | 0.0009 | | 0.9967 | | ENSG00000212916 | |  |
| SLC2A3 | -0.58 | | 0.0010 | | 0.9967 | | ENSG00000059804 | |  |
| ANKRD37 | -0.66 | | 0.0010 | | 0.9967 | | ENSG00000186352 | |  |
| ERRFI1 | -0.62 | | 0.0010 | | 0.9967 | | ENSG00000116285 | |  |
| NA | 0.65 | | 0.0011 | | 0.9967 | | ENSG00000253508 | |  |
| RASSF9 | 0.65 | | 0.0011 | | 0.9967 | | ENSG00000198774 | |  |
| JOSD1 | -0.24 | | 0.0012 | | 0.9967 | | ENSG00000100221 | |  |
| AQP9 | -0.65 | | 0.0012 | | 0.9967 | | ENSG00000103569 | |  |
| SLC6A19 | 0.48 | | 0.0012 | | 0.9967 | | ENSG00000174358 | |  |
| OSM | -0.65 | | 0.0012 | | 0.9967 | | ENSG00000099985 | |  |
| AIFM3 | -0.62 | | 0.0014 | | 0.9967 | | ENSG00000183773 | |  |
| LGI2 | 0.45 | | 0.0014 | | 0.9967 | | ENSG00000153012 | |  |
| SLC24A4 | -0.63 | | 0.0014 | | 0.9967 | | ENSG00000140090 | |  |
| LAMA3 | -0.64 | | 0.0014 | | 0.9967 | | ENSG00000053747 | |  |
| MYC | -0.52 | | 0.0015 | | 0.9967 | | ENSG00000136997 | |  |
| ALDH1A2 | -0.62 | | 0.0016 | | 0.9967 | | ENSG00000128918 | |  |
| USP51 | 0.46 | | 0.0016 | | 0.9967 | | ENSG00000247746 | |  |
| KCNH7 | 0.63 | | 0.0016 | | 0.9967 | | ENSG00000184611 | |  |
| MTERF1 | 0.26 | | 0.0017 | | 0.9967 | | ENSG00000127989 | |  |
| OXTR | -0.63 | | 0.0017 | | 0.9967 | | ENSG00000180914 | |  |
| C3 | -0.63 | | 0.0017 | | 0.9967 | | ENSG00000125730 | |  |
| MCL1 | -0.25 | | 0.0018 | | 0.9967 | | ENSG00000143384 | |  |
| AGPAT5 | 0.60 | | 0.0018 | | 0.9967 | | ENSG00000155189 | |  |
| PROSER2-AS1 | -0.48 | | 0.0018 | | 0.9967 | | ENSG00000225778 | |  |
| APOLD1 | -0.62 | | 0.0018 | | 0.9967 | | ENSG00000178878 | |  |
| SKIDA1 | 0.42 | | 0.0018 | | 0.9967 | | ENSG00000180592 | |  |
| LINC00162 | -0.59 | | 0.0019 | | 0.9967 | | ENSG00000275874 | |  |
| SRF | -0.44 | | 0.0019 | | 0.9967 | | ENSG00000112658 | |  |
| BHLHE41 | 0.52 | | 0.0020 | | 0.9967 | | ENSG00000123095 | |  |
| TDRD9 | -0.60 | | 0.0020 | | 0.9967 | | ENSG00000156414 | |  |
| STON2 | 0.53 | | 0.0020 | | 0.9967 | | ENSG00000140022 | |  |
| NA | 0.61 | | 0.0020 | | 0.9967 | | ENSG00000266903 | |  |
| MUC1 | -0.61 | | 0.0021 | | 0.9967 | | ENSG00000185499 | |  |
| FSTL3 | -0.61 | | 0.0023 | | 0.9967 | | ENSG00000070404 | |  |
| CXCL3 | -0.59 | | 0.0023 | | 0.9967 | | ENSG00000163734 | |  |
| NA | -0.59 | | 0.0023 | | 0.9967 | | ENSG00000238290 | |  |
| NA | 0.42 | | 0.0023 | | 0.9967 | | ENSG00000224992 | |  |
| NBPF26 | -0.59 | | 0.0023 | | 0.9967 | | ENSG00000273136 | |  |
| KCNJ16 | -0.60 | | 0.0025 | | 0.9967 | | ENSG00000153822 | |  |
| NOXRED1 | 0.56 | | 0.0025 | | 0.9967 | | ENSG00000165555 | |  |
| HCAR3 | -0.60 | | 0.0026 | | 0.9967 | | ENSG00000255398 | |  |
| NAT8B | 0.59 | | 0.0026 | | 0.9967 | | ENSG00000204872 | |  |
| SLC25A25 | -0.38 | | 0.0026 | | 0.9967 | | ENSG00000148339 | |  |

Supplemental Table 3C. List of the 70 DEGs from the placenta investigating fetal variables sorted on FDR and unadjusted p-values.

**Offspring sex**. girls/boys

| **symbol** | **log2FC** | **p-value** | **p-adjust.** | **Ensembl** |
| --- | --- | --- | --- | --- |
| RPS4Y1 | 7.03 | 0.00E+00 | 0 | ENSG00000129824 |
| USP9Y | 5.95 | 0.00E+00 | 0 | ENSG00000114374 |
| DDX3Y | 6.92 | 0.00E+00 | 0 | ENSG00000067048 |
| UTY | 5.80 | 0.00E+00 | 0 | ENSG00000183878 |
| KDM5D | 5.96 | 0.00E+00 | 0 | ENSG00000012817 |
| EIF1AY | 5.68 | 0.00E+00 | 0 | ENSG00000198692 |
| ZFY | 5.47 | 2.72E-291 | 1.11E-287 | ENSG00000067646 |
| TSIX | -2.29 | 5.24E-42 | 1.15E-38 | ENSG00000270641 |
| TXLNGY | 3.80 | 4.67E-115 | 1.34E-111 | ENSG00000131002 |
| EIF2S3 | -0.34 | 1.36E-09 | 1.44E-06 | ENSG00000130741 |
| VAMP7 | 0.43 | 1.83E-08 | 1.75E-05 | ENSG00000124333 |
| NA | 1.39 | 1.42E-16 | 1.93E-13 | ENSG00000273906 |
| TMSB4Y | 1.63 | 1.65E-21 | 2.35E-18 | ENSG00000154620 |
| PCDH11X | 2.38 | 1.21E-44 | 3.14E-41 | ENSG00000099715 |
| GYG2P1 | 2.22 | 1.72E-38 | 3.27E-35 | ENSG00000206159 |
| HDHD1 | -0.79 | 3.36E-10 | 3.70E-07 | ENSG00000130021 |
| NA | 1.05 | 3.91E-11 | 4.65E-08 | ENSG00000230663 |
| NA | 0.83 | 5.49E-08 | 5.06E-05 | ENSG00000228786 |
| NA | 1.65 | 3.49E-22 | 5.26E-19 | ENSG00000229308 |
| KDM6A | -0.66 | 4.29E-12 | 5.57E-09 | ENSG00000147050 |
| NA | 0.90 | 5.63E-09 | 5.75E-06 | ENSG00000278847 |
| NA | 1.92 | 3.48E-29 | 5.86E-26 | ENSG00000233070 |
| NA | 1.05 | 5.13E-11 | 5.86E-08 | ENSG00000241859 |
| LINC00278 | 1.81 | 3.75E-26 | 5.96E-23 | ENSG00000231535 |
| XIST | -2.25 | 3.14E-39 | 6.42E-36 | ENSG00000229807 |
| DDX3X | -0.50 | 6.85E-09 | 6.76E-06 | ENSG00000215301 |
| NLGN4Y | 1.07 | 6.67E-12 | 8.29E-09 | ENSG00000165246 |
| TTTY14 | 2.18 | 5.29E-37 | 9.45E-34 | ENSG00000176728 |
| TTTY15 | 4.83 | 2.66E-219 | 9.52E-216 | ENSG00000233864 |
| NA | 2.35 | 4.09E-43 | 9.74E-40 | ENSG00000260197 |
| PRKY | 3.86 | 3.15E-120 | 9.99E-117 | ENSG00000099725 |
| ZFX | -0.40 | 1.30E-07 | 0.0001 | ENSG00000005889 |
| NA | 0.81 | 1.44E-07 | 0.0001 | ENSG00000278212 |
| EIF1AX | -0.45 | 2.70E-06 | 0.0023 | ENSG00000173674 |
| BRF2 | -0.26 | 1.06E-05 | 0.0087 | ENSG00000104221 |
| NA | 0.60 | 1.15E-05 | 0.0091 | ENSG00000237659 |
| MIAT | 0.70 | 1.59E-05 | 0.0123 | ENSG00000225783 |
| TBL1Y | 0.56 | 2.79E-05 | 0.0210 | ENSG00000092377 |
| KDM5C | -0.50 | 3.63E-05 | 0.0266 | ENSG00000126012 |
| NA | 0.54 | 4.49E-05 | 0.0321 | ENSG00000267793 |
| NA | 0.59 | 6.21E-05 | 0.0433 | ENSG00000211598 |
| SMC1A | -0.42 | 6.66E-05 | 0.0453 | ENSG00000072501 |
| SLFN12L | 0.66 | 9.26E-05 | 0.0615 | ENSG00000205045 |
| HSD17B10 | -0.34 | 1.08E-04 | 0.0686 | ENSG00000072506 |
| NA | 0.66 | 1.06E-04 | 0.0686 | ENSG00000268439 |
| CXCL10 | 0.64 | 1.20E-04 | 0.0743 | ENSG00000169245 |
| TTTY10 | 0.49 | 1.27E-04 | 0.0771 | ENSG00000229236 |
| IGJ | 0.61 | 1.34E-04 | 0.0780 | ENSG00000132465 |
| NA | 0.64 | 1.33E-04 | 0.0780 | ENSG00000211893 |
| CCDC51 | -0.38 | 1.48E-04 | 0.0844 | ENSG00000164051 |
| PRAME | -0.57 | 1.73E-04 | 0.0970 | ENSG00000185686 |
| NA | 0.62 | 2.45E-04 | 0.1333 | ENSG00000211897 |
| NA | 0.45 | 2.47E-04 | 0.1333 | ENSG00000229163 |
| HDAC8 | -0.32 | 2.59E-04 | 0.1371 | ENSG00000147099 |
| NA | 0.45 | 2.67E-04 | 0.1386 | ENSG00000235462 |
| NA | 0.59 | 3.15E-04 | 0.1581 | ENSG00000227603 |
| MYO1A | -0.58 | 3.13E-04 | 0.1581 | ENSG00000166866 |
| HILPDA | 0.61 | 3.75E-04 | 0.1847 | ENSG00000135245 |
| CXCL9 | 0.53 | 4.05E-04 | 0.1920 | ENSG00000138755 |
| LINC01510 | -0.51 | 4.15E-04 | 0.1920 | ENSG00000231210 |
| STX16 | 0.18 | 4.04E-04 | 0.1920 | ENSG00000124222 |
| NA | 0.45 | 4.16E-04 | 0.1920 | ENSG00000211677 |
| DNAJC5B | 0.44 | 4.82E-04 | 0.2187 | ENSG00000147570 |
| CD3D | 0.59 | 5.11E-04 | 0.2282 | ENSG00000167286 |
| CD8A | 0.59 | 5.96E-04 | 0.2562 | ENSG00000153563 |
| NA | 0.46 | 6.00E-04 | 0.2562 | ENSG00000241351 |
| NA | 0.57 | 5.84E-04 | 0.2562 | ENSG00000275055 |
| LAS1L | -0.24 | 6.23E-04 | 0.2620 | ENSG00000001497 |
| NA | 0.42 | 6.65E-04 | 0.2755 | ENSG00000255197 |
| MIR6895 | -0.54 | 6.94E-04 | 0.2793 | ENSG00000276575 |

Placenta weight

| **symbol** | **log2FC** | **p-value** | **p-adjusted** | **Ensembl** |
| --- | --- | --- | --- | --- |
| HBD | 0.76 | 1.88E-05 | 0.0981 | ENSG00000223609 |
| RHCE | 0.75 | 1.98E-05 | 0.0981 | ENSG00000188672 |
| SUPT3H | -0.34 | 2.21E-05 | 0.0981 | ENSG00000196284 |
| TMCC2 | 0.76 | 9.51E-06 | 0.0981 | ENSG00000133069 |
| TRIM10 | 0.75 | 3.43E-05 | 0.1215 | ENSG00000204613 |
| FAM46C | 0.68 | 4.17E-05 | 0.1233 | ENSG00000183508 |
| ACSL6 | 0.68 | 7.94E-05 | 0.1928 | ENSG00000164398 |
| SLC4A1 | 0.69 | 9.75E-05 | 0.1928 | ENSG00000004939 |
| HBB | 0.70 | 9.78E-05 | 0.1928 | ENSG00000244734 |
| NLRP3 | 0.49 | 0.0001 | 0.2182 | ENSG00000162711 |
| AHSP | 0.69 | 0.0001 | 0.2218 | ENSG00000169877 |
| EPB42 | 0.67 | 0.0002 | 0.2876 | ENSG00000166947 |
| OSBP2 | 0.56 | 0.0002 | 0.3089 | ENSG00000184792 |
| EPB41 | 0.43 | 0.0003 | 0.3089 | ENSG00000159023 |
| GYPA | 0.65 | 0.0003 | 0.3089 | ENSG00000170180 |
| IFIT1B | 0.65 | 0.0003 | 0.3216 | ENSG00000204010 |
| KRT1 | 0.65 | 0.0003 | 0.3516 | ENSG00000167768 |
| NA | 0.59 | 0.0004 | 0.4391 | ENSG00000277639 |
| SPI1 | 0.35 | 0.0005 | 0.4522 | ENSG00000066336 |
| CHAF1B | -0.39 | 0.0006 | 0.4812 | ENSG00000159259 |
| CXCL13 | 0.54 | 0.0006 | 0.4812 | ENSG00000156234 |
| AGR2 | 0.61 | 0.0006 | 0.4812 | ENSG00000106541 |
| CRYBB1 | 0.47 | 0.0007 | 0.4812 | ENSG00000100122 |
| ZBP1 | 0.60 | 0.0007 | 0.4812 | ENSG00000124256 |
| ALAS2 | 0.61 | 0.0007 | 0.4812 | ENSG00000158578 |
| STAC | 0.58 | 0.0009 | 0.5857 | ENSG00000144681 |
| HSD3B2 | 0.58 | 0.0010 | 0.5857 | ENSG00000203859 |
| XK | 0.50 | 0.0010 | 0.5857 | ENSG00000047597 |
| CORO1A | 0.42 | 0.0010 | 0.5857 | ENSG00000102879 |
| TAC3 | 0.59 | 0.0011 | 0.5857 | ENSG00000166863 |
| CCNA2 | -0.37 | 0.0011 | 0.5857 | ENSG00000145386 |
| PRR9 | -0.58 | 0.0011 | 0.5857 | ENSG00000203783 |
| HYKK | -0.51 | 0.0012 | 0.5857 | ENSG00000188266 |
| CD2 | 0.58 | 0.0012 | 0.5857 | ENSG00000116824 |
| DPYD | 0.47 | 0.0012 | 0.5857 | ENSG00000188641 |
| SELENBP1 | 0.49 | 0.0012 | 0.5857 | ENSG00000143416 |
| SLC25A37 | 0.43 | 0.0013 | 0.5857 | ENSG00000147454 |
| HEMGN | 0.58 | 0.0013 | 0.5857 | ENSG00000136929 |
| PCDHB5 | -0.58 | 0.0013 | 0.5857 | ENSG00000113209 |
| CCL4L2 | 0.57 | 0.0013 | 0.5857 | ENSG00000276070 |
| AOAH | 0.41 | 0.0015 | 0.6278 | ENSG00000136250 |
| CCR5 | 0.57 | 0.0015 | 0.6520 | ENSG00000160791 |
| C21orf33 | -0.56 | 0.0016 | 0.6609 | ENSG00000160221 |
| CCL4 | 0.56 | 0.0018 | 0.7115 | ENSG00000275302 |
| RHAG | 0.54 | 0.0018 | 0.7161 | ENSG00000112077 |
| GYPB | 0.56 | 0.0019 | 0.7485 | ENSG00000250361 |
| PDE8A | -0.26 | 0.0020 | 0.7562 | ENSG00000073417 |
| DYNC1I1 | 0.54 | 0.0021 | 0.7562 | ENSG00000158560 |
| CD38 | 0.55 | 0.0021 | 0.7562 | ENSG00000004468 |
| CLEC10A | 0.55 | 0.0021 | 0.7562 | ENSG00000132514 |
| COL21A1 | -0.48 | 0.0024 | 0.8363 | ENSG00000124749 |
| TAPBPL | 0.41 | 0.0025 | 0.8375 | ENSG00000139192 |
| SLC15A1 | 0.54 | 0.0026 | 0.8375 | ENSG00000088386 |
| CD83 | 0.53 | 0.0026 | 0.8375 | ENSG00000112149 |
| SNCA | 0.53 | 0.0026 | 0.8375 | ENSG00000145335 |
| CD300C | 0.48 | 0.0027 | 0.8375 | ENSG00000167850 |
| NA | 0.53 | 0.0027 | 0.8375 | ENSG00000260592 |
| HBG2 | 0.54 | 0.0028 | 0.8497 | ENSG00000196565 |
| GCK | 0.51 | 0.0029 | 0.8497 | ENSG00000106633 |
| CTSS | 0.44 | 0.0029 | 0.8497 | ENSG00000163131 |
| DEFA4 | 0.49 | 0.0030 | 0.8497 | ENSG00000164821 |
| FIRRE | -0.41 | 0.0030 | 0.8497 | ENSG00000213468 |
| NA | 0.53 | 0.0030 | 0.8497 | ENSG00000263082 |
| C3AR1 | 0.34 | 0.0031 | 0.8497 | ENSG00000171860 |
| CCL3L1 | 0.52 | 0.0031 | 0.8572 | ENSG00000276085 |
| TMIGD2 | 0.51 | 0.0032 | 0.8644 | ENSG00000167664 |
| SLC23A3 | -0.48 | 0.0033 | 0.8828 | ENSG00000213901 |
| CD53 | 0.34 | 0.0035 | 0.9140 | ENSG00000143119 |
| RTN4RL1 | 0.51 | 0.0036 | 0.9140 | ENSG00000185924 |
| ORM1 | 0.50 | 0.0036 | 0.9140 | ENSG00000229314 |

**Supplemental Table 4.** List of the 70 DEGs from the placenta samples between PE and control and GDM and control sorted on FDR and unadjusted p-values.

**Gestational diabetes mellitus**

| **symbol** | **log2FC** | **p-value** | **p-adjust.** | **Ensembl** |
| --- | --- | --- | --- | --- |
| GH1 | -0.80 | 5.23E-05 | 0.6630 | ENSG00000259384 |
| LOC101928915 | 0.71 | 5.33E-05 | 0.6630 | ENSG00000224746 |
| HSD3B2 | 0.77 | 5.49E-05 | 0.6630 | ENSG00000203859 |
| TIGD4 | -0.77 | 0.0001 | 0.9864 | ENSG00000169989 |
| SYT6 | 0.71 | 0.0002 | 0.9953 | ENSG00000134207 |
| NA | 0.70 | 0.0003 | 0.9953 | ENSG00000272156 |
| ACKR1 | 0.70 | 0.0003 | 0.9953 | ENSG00000213088 |
| MYH13 | 0.71 | 0.0004 | 0.9953 | ENSG00000006788 |
| NA | -0.65 | 0.0005 | 0.9953 | ENSG00000267312 |
| NA | -0.66 | 0.0006 | 0.9953 | ENSG00000260774 |
| FGF12 | -0.65 | 0.0007 | 0.9953 | ENSG00000114279 |
| CDH15 | 0.67 | 0.0008 | 0.9953 | ENSG00000129910 |
| CFD | 0.67 | 0.0008 | 0.9953 | ENSG00000197766 |
| CRHBP | 0.65 | 0.0009 | 0.9953 | ENSG00000145708 |
| FZD2 | 0.55 | 0.0009 | 0.9953 | ENSG00000180340 |
| EFNA5 | 0.56 | 0.0010 | 0.9953 | ENSG00000184349 |
| PKIA | -0.64 | 0.0010 | 0.9953 | ENSG00000171033 |
| ADGRE3 | -0.65 | 0.0011 | 0.9953 | ENSG00000131355 |
| PROK2 | -0.64 | 0.0012 | 0.9953 | ENSG00000163421 |
| NA | -0.55 | 0.0012 | 0.9953 | ENSG00000232053 |
| NA | 0.62 | 0.0012 | 0.9953 | ENSG00000277048 |
| NA | -0.64 | 0.0013 | 0.9953 | ENSG00000279748 |
| CXCL14 | 0.61 | 0.0014 | 0.9953 | ENSG00000145824 |
| HAPLN2 | 0.54 | 0.0016 | 0.9953 | ENSG00000132702 |
| CXCL10 | 0.62 | 0.0017 | 0.9953 | ENSG00000169245 |
| PROM1 | 0.41 | 0.0018 | 0.9953 | ENSG00000007062 |
| COL8A2 | 0.49 | 0.0019 | 0.9953 | ENSG00000171812 |
| GP6 | -0.61 | 0.0020 | 0.9953 | ENSG00000088053 |
| ZDHHC8P1 | 0.51 | 0.0020 | 0.9953 | ENSG00000133519 |
| HDC | 0.61 | 0.0020 | 0.9953 | ENSG00000140287 |
| ITGBL1 | 0.50 | 0.0021 | 0.9953 | ENSG00000198542 |
| ZNF786 | 0.41 | 0.0022 | 0.9953 | ENSG00000197362 |
| CADM3 | 0.57 | 0.0023 | 0.9953 | ENSG00000162706 |
| OLFML3 | 0.44 | 0.0025 | 0.9953 | ENSG00000116774 |
| SLC7A10 | -0.53 | 0.0025 | 0.9953 | ENSG00000130876 |
| CXCR2 | -0.60 | 0.0025 | 0.9953 | ENSG00000180871 |
| LOC100507477 | -0.59 | 0.0026 | 0.9953 | ENSG00000236013 |
| ENPP2 | 0.46 | 0.0027 | 0.9953 | ENSG00000136960 |
| ADGRE2 | -0.52 | 0.0028 | 0.9953 | ENSG00000127507 |
| NA | -0.42 | 0.0029 | 0.9953 | ENSG00000259959 |
| NA | 0.53 | 0.0029 | 0.9953 | ENSG00000280152 |
| MIR4258 | -0.43 | 0.0031 | 0.9953 | ENSG00000264349 |
| SCARF2 | 0.52 | 0.0031 | 0.9953 | ENSG00000244486 |
| BEGAIN | 0.54 | 0.0032 | 0.9953 | ENSG00000183092 |
| NA | -0.53 | 0.0036 | 0.9953 | ENSG00000248719 |
| MALRD1 | 0.57 | 0.0036 | 0.9953 | ENSG00000204740 |
| FAM83F | 0.58 | 0.0037 | 0.9953 | ENSG00000133477 |
| NA | 0.55 | 0.0037 | 0.9953 | ENSG00000279656 |
| NA | -0.55 | 0.0038 | 0.9953 | ENSG00000261729 |
| GDF6 | 0.51 | 0.0038 | 0.9953 | ENSG00000156466 |
| CYP24A1 | 0.56 | 0.0038 | 0.9953 | ENSG00000019186 |
| SAXO2 | 0.52 | 0.0041 | 0.9953 | ENSG00000188659 |
| LOC101928118 | 0.50 | 0.0042 | 0.9953 | ENSG00000226026 |
| DDX60L | 0.37 | 0.0043 | 0.9953 | ENSG00000181381 |
| CXCL9 | 0.52 | 0.0043 | 0.9953 | ENSG00000138755 |
| NA | -0.54 | 0.0044 | 0.9953 | ENSG00000267701 |
| NA | 0.44 | 0.0044 | 0.9953 | ENSG00000241351 |
| OSBPL6 | 0.55 | 0.0045 | 0.9953 | ENSG00000079156 |
| NA | 0.48 | 0.0047 | 0.9953 | ENSG00000225111 |
| NMB | 0.55 | 0.0047 | 0.9953 | ENSG00000197696 |
| NA | 0.56 | 0.0047 | 0.9953 | ENSG00000260459 |
| VASN | 0.53 | 0.0049 | 0.9953 | ENSG00000168140 |
| UBA7 | 0.37 | 0.0049 | 0.9953 | ENSG00000182179 |
| MUC4 | -0.53 | 0.0050 | 0.9953 | ENSG00000145113 |
| WNT6 | 0.56 | 0.0050 | 0.9953 | ENSG00000115596 |
| COL16A1 | 0.48 | 0.0050 | 0.9953 | ENSG00000084636 |
| FBXW9 | 0.34 | 0.0051 | 0.9953 | ENSG00000132004 |
| ANGPTL7 | 0.54 | 0.0051 | 0.9953 | ENSG00000171819 |
| ACCS | 0.52 | 0.0051 | 0.9953 | ENSG00000110455 |
| FCGR3B | -0.55 | 0.0052 | 0.9953 | ENSG00000162747 |

Pre-eclampsia

| **symbol** | **log2FC** | **p-value** | **p-adjust.** | **Ensembl** |
| --- | --- | --- | --- | --- |
| NA | -0.84 | 4.03E-08 | 0.0007 | ENSG00000262831 |
| ZDHHC8P1 | 0.71 | 1.93E-07 | 0.0016 | ENSG00000133519 |
| ANGPT2 | -0.88 | 1.14E-05 | 0.0638 | ENSG00000091879 |
| NA | -0.90 | 1.50E-05 | 0.0638 | ENSG00000249898 |
| LOC100506321 | -0.87 | 2.68E-05 | 0.0915 | ENSG00000259118 |
| NA | -0.79 | 4.43E-05 | 0.1090 | ENSG00000234477 |
| FAM184A | 0.57 | 0.0001 | 0.1090 | ENSG00000111879 |
| ISL1 | 0.67 | 0.0001 | 0.1090 | ENSG00000016082 |
| NPDC1 | 0.69 | 0.0001 | 0.1090 | ENSG00000107281 |
| ID1 | 0.71 | 0.0001 | 0.1090 | ENSG00000125968 |
| STMN3 | 0.52 | 0.0001 | 0.1090 | ENSG00000197457 |
| REEP2 | 0.79 | 0.0001 | 0.1090 | ENSG00000132563 |
| CCDC85C | 0.46 | 0.0001 | 0.1289 | ENSG00000205476 |
| ID3 | 0.52 | 0.0001 | 0.1289 | ENSG00000117318 |
| ENKD1 | 0.43 | 0.0001 | 0.1331 | ENSG00000124074 |
| HSPA1A | 0.74 | 0.0002 | 0.1623 | ENSG00000204389 |
| SLC38A3 | 0.74 | 0.0002 | 0.1623 | ENSG00000188338 |
| NA | -0.76 | 0.0002 | 0.1713 | ENSG00000267601 |
| AGPAT5 | -0.75 | 0.0002 | 0.1713 | ENSG00000155189 |
| JPH1 | 0.78 | 0.0002 | 0.1713 | ENSG00000104369 |
| PROM1 | 0.52 | 0.0002 | 0.1713 | ENSG00000007062 |
| JAG2 | 0.62 | 0.0002 | 0.1786 | ENSG00000184916 |
| ABI3 | 0.55 | 0.0002 | 0.1852 | ENSG00000108798 |
| IER5L | 0.76 | 0.0003 | 0.1852 | ENSG00000188483 |
| SNX22 | -0.64 | 0.0003 | 0.1852 | ENSG00000157734 |
| SSR3 | -0.30 | 0.0003 | 0.1852 | ENSG00000114850 |
| RBM3 | -0.39 | 0.0003 | 0.1951 | ENSG00000102317 |
| NA | -0.65 | 0.0003 | 0.1951 | ENSG00000281415 |
| BEGAIN | 0.72 | 0.0003 | 0.1951 | ENSG00000183092 |
| SH3RF3 | 0.51 | 0.0004 | 0.2112 | ENSG00000172985 |
| VEGFC | 0.63 | 0.0004 | 0.2112 | ENSG00000150630 |
| CARD10 | 0.58 | 0.0004 | 0.2112 | ENSG00000100065 |
| LOC100507334 | 0.73 | 0.0004 | 0.2112 | ENSG00000261760 |
| MFSD4 | 0.66 | 0.0004 | 0.2152 | ENSG00000174514 |
| NA | -0.74 | 0.0004 | 0.2152 | ENSG00000229893 |
| SLC27A6 | -0.52 | 0.0005 | 0.2152 | ENSG00000113396 |
| NOTCH1 | 0.58 | 0.0005 | 0.2208 | ENSG00000148400 |
| PAWR | -0.46 | 0.0005 | 0.2208 | ENSG00000177425 |
| MOXD1 | -0.69 | 0.0005 | 0.2208 | ENSG00000079931 |
| CLDN5 | 0.65 | 0.0005 | 0.2208 | ENSG00000184113 |
| CXCL14 | 0.63 | 0.0006 | 0.2208 | ENSG00000145824 |
| DUSP6 | 0.46 | 0.0006 | 0.2208 | ENSG00000139318 |
| CRTC1 | 0.44 | 0.0006 | 0.2208 | ENSG00000105662 |
| EGFL7 | 0.57 | 0.0006 | 0.2208 | ENSG00000172889 |
| NRGN | 0.59 | 0.0006 | 0.2208 | ENSG00000154146 |
| SOX18 | 0.68 | 0.0006 | 0.2208 | ENSG00000203883 |
| NA | -0.66 | 0.0006 | 0.2208 | ENSG00000275481 |
| LOC100507477 | -0.72 | 0.0006 | 0.2259 | ENSG00000236013 |
| COL9A2 | 0.60 | 0.0007 | 0.2333 | ENSG00000049089 |
| SEC11C | -0.49 | 0.0007 | 0.2422 | ENSG00000166562 |
| GALNT18 | 0.52 | 0.0007 | 0.2428 | ENSG00000110328 |
| PPP2R5B | 0.38 | 0.0007 | 0.2428 | ENSG00000068971 |
| MCF2L | 0.69 | 0.0008 | 0.2463 | ENSG00000126217 |
| SUMF2 | -0.51 | 0.0008 | 0.2463 | ENSG00000129103 |
| CASZ1 | 0.51 | 0.0008 | 0.2463 | ENSG00000130940 |
| TAF1B | -0.47 | 0.0008 | 0.2463 | ENSG00000115750 |
| ERP27 | -0.69 | 0.0009 | 0.2463 | ENSG00000139055 |
| SUCO | -0.30 | 0.0009 | 0.2463 | ENSG00000094975 |
| ABHD17A | 0.41 | 0.0009 | 0.2463 | ENSG00000129968 |
| KCTD15 | 0.57 | 0.0009 | 0.2463 | ENSG00000153885 |
| FAM213B | 0.57 | 0.0009 | 0.2463 | ENSG00000157870 |
| NA | 0.66 | 0.0009 | 0.2558 | ENSG00000266176 |
| MN1 | 0.55 | 0.0010 | 0.2558 | ENSG00000169184 |
| TNFRSF10D | 0.54 | 0.0010 | 0.2558 | ENSG00000173530 |
| NA | -0.58 | 0.0010 | 0.2558 | ENSG00000267469 |
| ATP6V1E2 | -0.52 | 0.0010 | 0.2558 | ENSG00000250565 |
| LGI4 | 0.69 | 0.0010 | 0.2558 | ENSG00000153902 |
| MIR126 | 0.55 | 0.0010 | 0.2558 | ENSG00000199161 |
| PIR | -0.43 | 0.0010 | 0.2558 | ENSG00000087842 |
| GABARAP | -0.36 | 0.0011 | 0.2558 | ENSG00000170296 |

Figure 1. Overview over the samples and methods used.


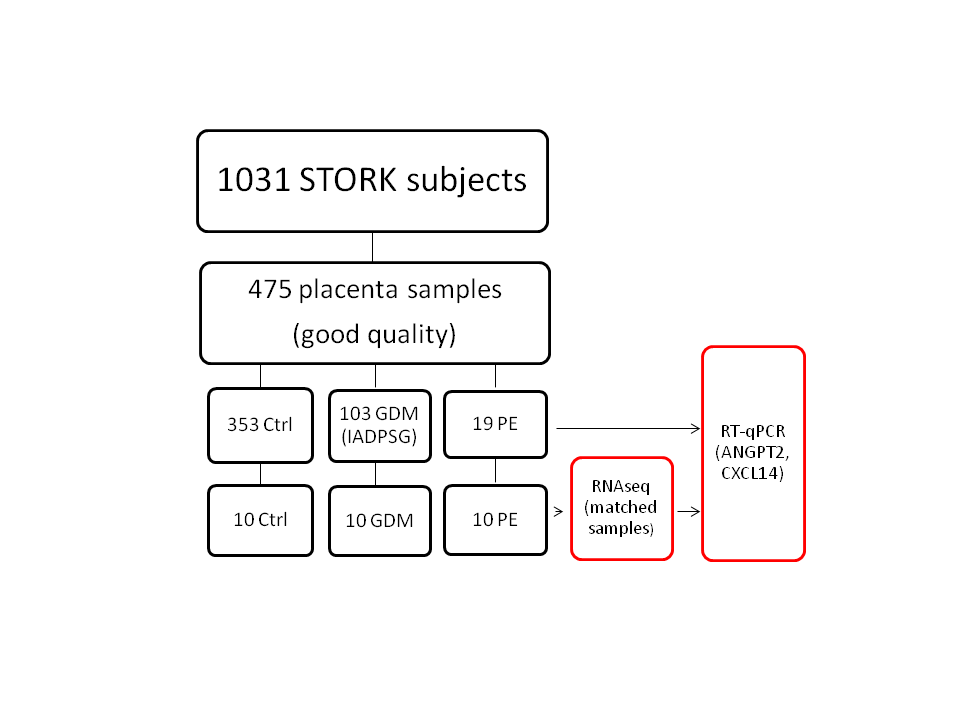


Figure 2A. Nanodrop and bioanalyzer analyses of samples chosen for RNAseq from placentas from controls, GDM and PE women. RIN values are shown at the top of the figure and absorbance 260/280 at the bottom of the figure. A ladder (L) showing the different peaks are included on every run.


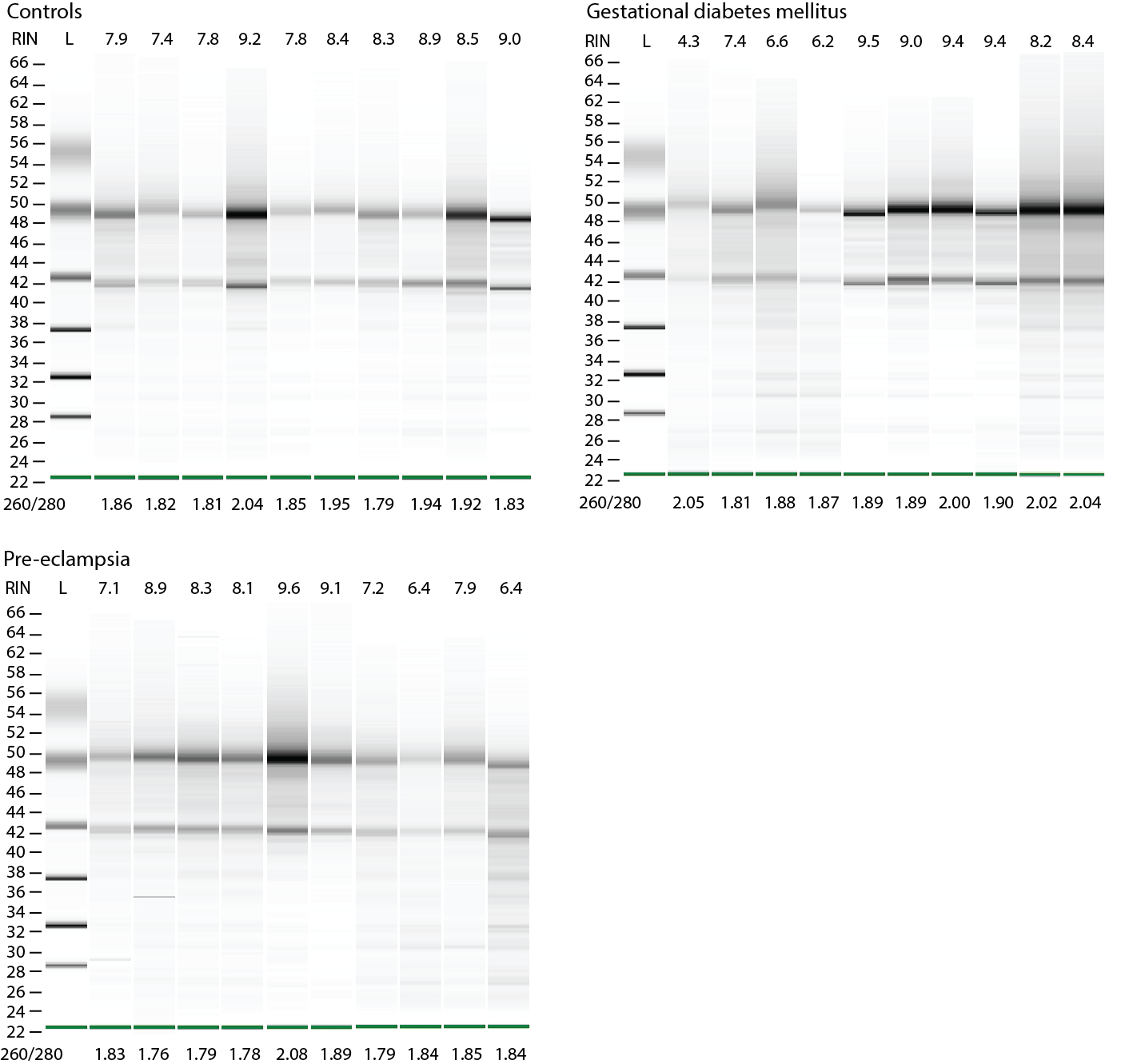


Figure 2B. Electropherogram from the bioanalyzer analysis of the samples chosen for RNAseq from placentas from controls, GDM and PE women.

Controls

1 2


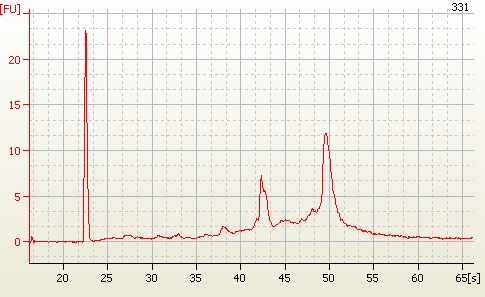

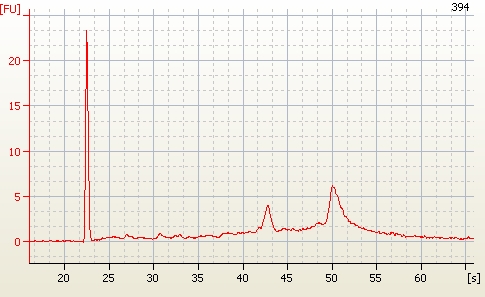


3 4


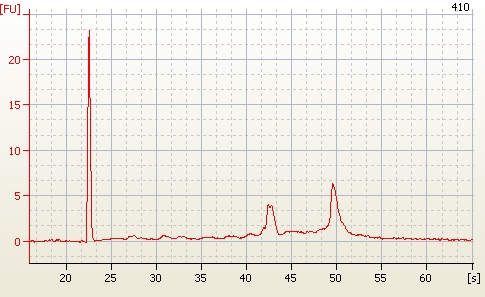

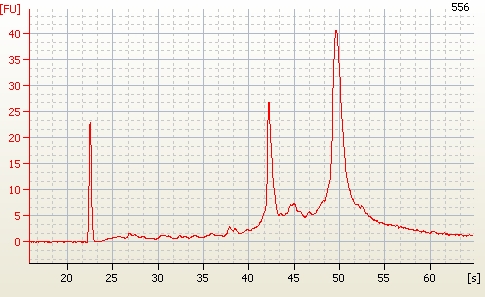


5 6


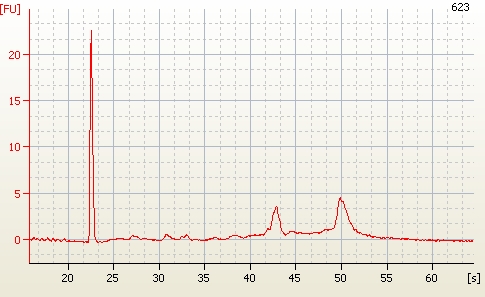

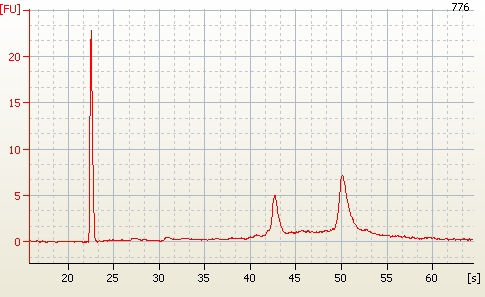


7 8


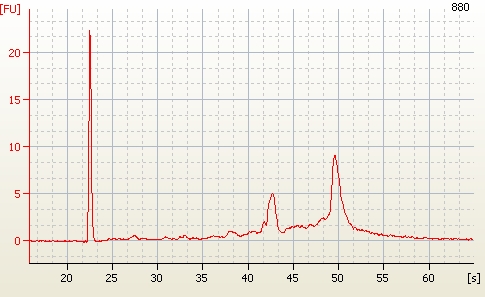

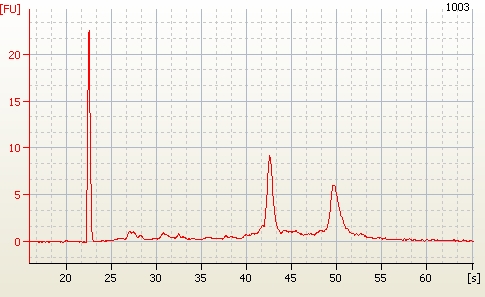


9 10


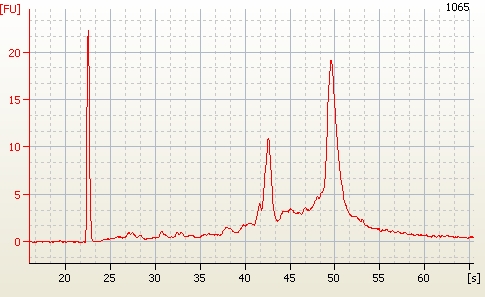

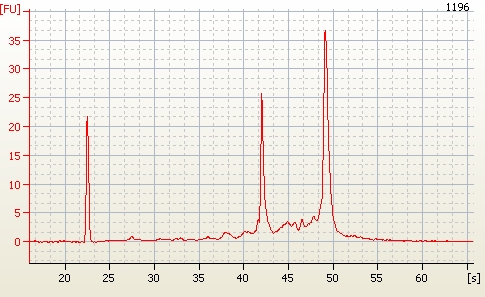


GDM

1 2


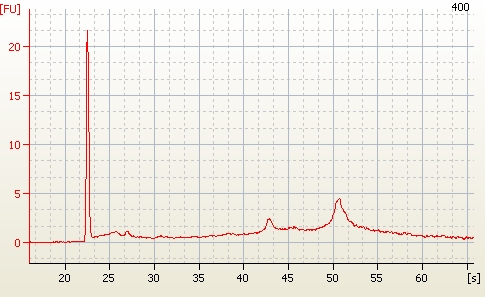

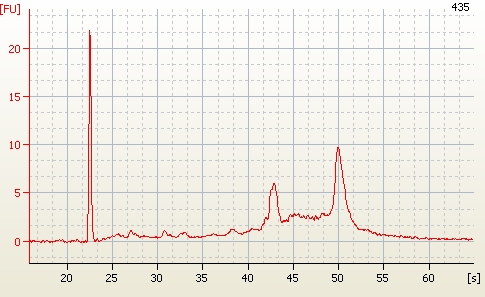


3 4


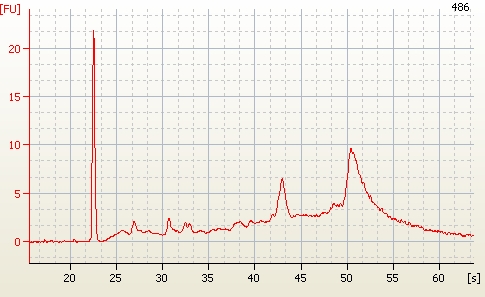

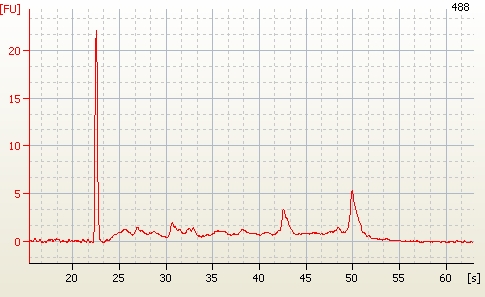


5 6


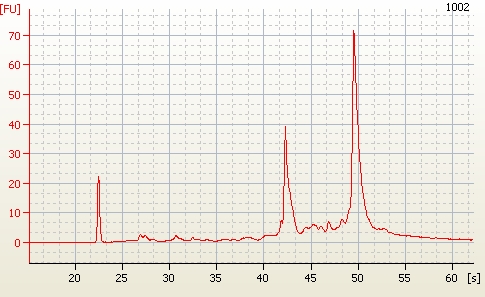

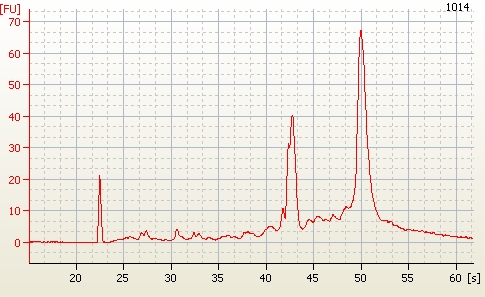


7 8


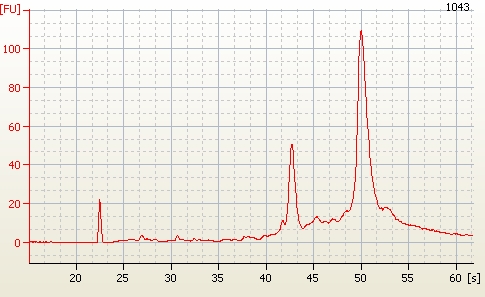

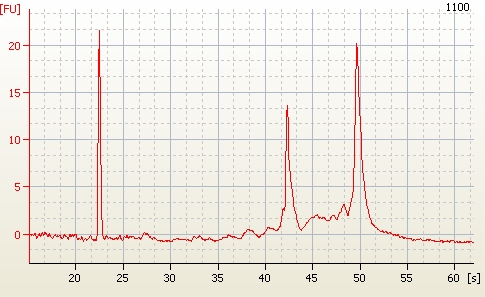


9 10


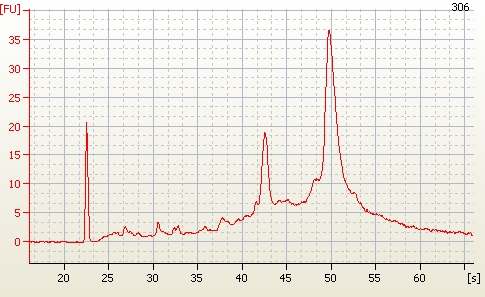

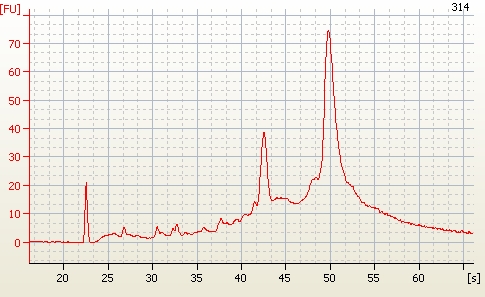


Pre-eclampsia

1 2


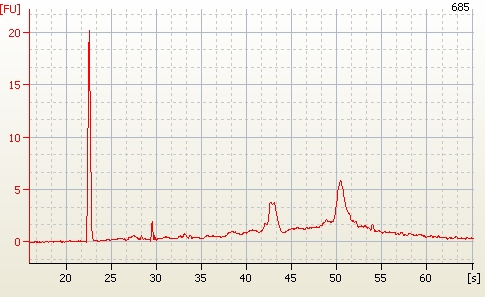

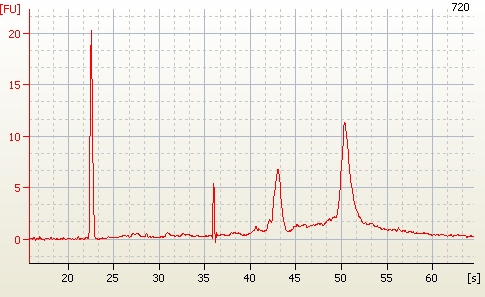


3 4


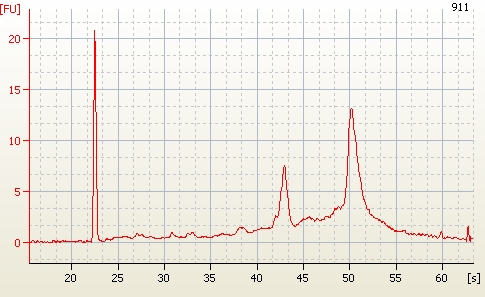

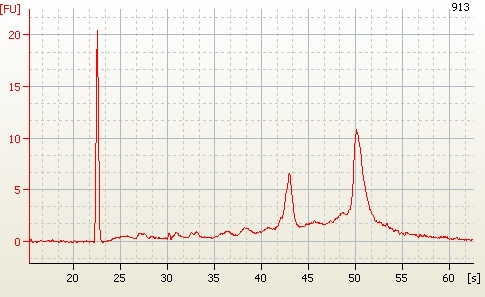


5 6


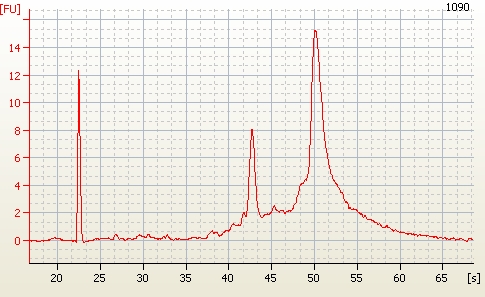

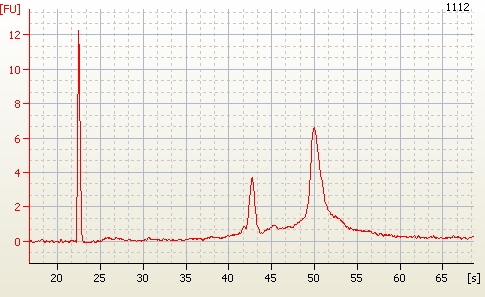


7 8


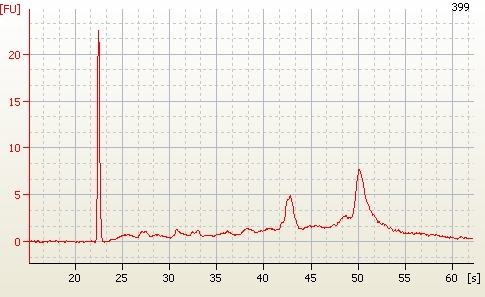

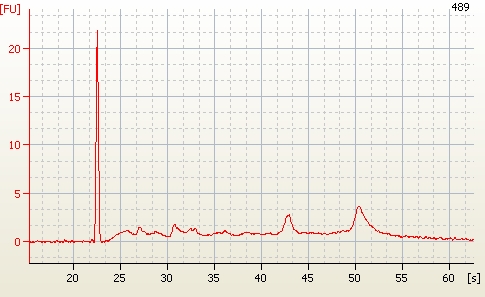


9 10


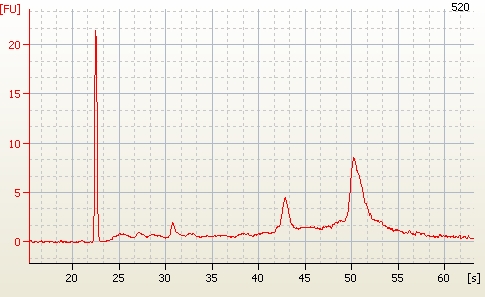

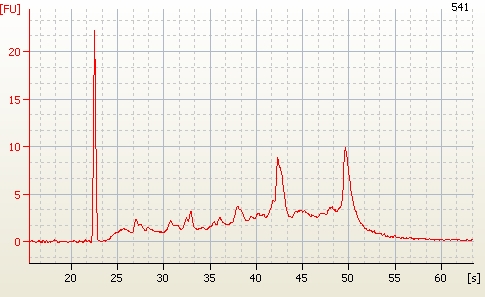


Figure 3. Expression levels of a) ANGPT2 and b) CXCL14 in the placenta between Ctrl (yellow), GDM (purple) and PE (red), with qPCR in the samples chosen for RNAseq (30 samples) and in the validation cohort (475 samples). Data is given as median and 25^th^/75^th^ percentile. *p<0.05 **p<0.01, compared with the control group.


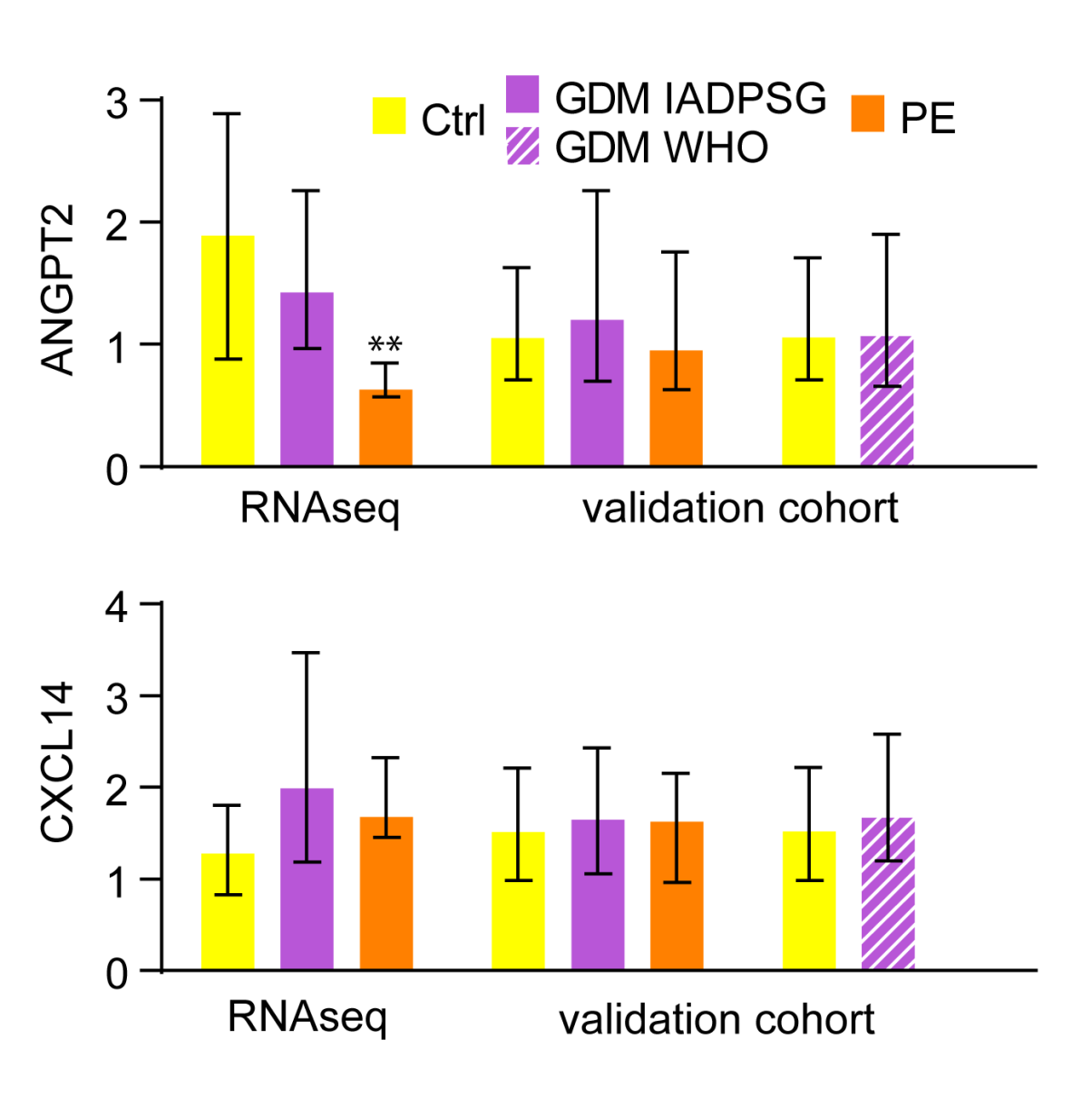

Supplement: Supplementary Information [file srep29715-s1.doc]
